# Anti-podocalyxin antibody exerts antitumor effects via antibody-dependent cellular cytotoxicity in mouse xenograft models of oral squamous cell carcinoma

**DOI:** 10.18632/oncotarget.25132

**Published:** 2018-04-27

**Authors:** Shunsuke Itai, Tomokazu Ohishi, Mika K. Kaneko, Shinji Yamada, Shinji Abe, Takuro Nakamura, Miyuki Yanaka, Yao-Wen Chang, Shun-Ichi Ohba, Yasuhiko Nishioka, Manabu Kawada, Hiroyuki Harada, Yukinari Kato

**Affiliations:** ^1^ Department of Antibody Drug Development, Tohoku University Graduate School of Medicine, Aoba-ku, Sendai, Miyagi 980-8575, Japan; ^2^ Department of Oral and Maxillofacial Surgery, Graduate School of Medical and Dental Sciences, Tokyo Medical and Dental University, Bunkyo-ku, Tokyo 113-8510, Japan; ^3^ Institute of Microbial Chemistry, BIKAKEN, Numazu, Microbial Chemistry Research Foundation, Numazu-shi, Shizuoka 410-0301, Japan; ^4^ Department of Clinical Pharmacy Practice Pedagogy, Graduate School of Biomedical Sciences, Tokushima University, Tokushima 770-8505, Japan; ^5^ Department of Respiratory Medicine and Rheumatology, Graduate School of Biomedical Sciences, Tokushima University, Tokushima 770-8503, Japan; ^6^ New Industry Creation Hatchery Center, Tohoku University, Aoba-ku, Sendai, Miyagi 980-8575, Japan

**Keywords:** podocalyxin, PODXL, monoclonal antibody, antibody-dependent cellular cytotoxicity, oral squamous cell carcinoma

## Abstract

Podocalyxin (PODXL) overexpression is associated with progression, metastasis, and poor outcomes in cancers. We recently produced the novel anti-PODXL monoclonal antibody (mAb) PcMab-47 (IgG_1_, kappa). Herein, we engineered PcMab-47 into 47-mG_2a_, a mouse IgG_2a_-type mAb, to add antibody-dependent cellular cytotoxicity (ADCC). We further developed 47-mG_2a_-f, a core fucose-deficient type of 47-mG_2a_ to augment its ADCC. Immunohistochemical analysis of oral cancer tissues using PcMab-47 and 47-mG_2a_ revealed that the latter stained oral squamous cell carcinoma (OSCC) cells in a cytoplasmic pattern at a much lower concentration. PcMab-47 and 47-mG_2a_ detected PODXL in 163/201 (81.1%) and in 197/201 (98.0%) OSCC samples, respectively. 47-mG_2a_-f also detected PODXL in OSCCs at a similar frequency as 47-mG_2a_. *In vitro* analysis revealed that both 47-mG_2a_ and 47-mG_2a_-f exhibited strong complement-dependent cytotoxicity (CDC) against CHO/hPODXL cells. In contrast, 47-mG_2a_-f exhibited much stronger ADCC than 47-mG_2a_ against OSCC cells, indicating that ADCC and CDC of those anti-PODXL mAbs depend on target cells. *In vivo* analysis revealed that both 47-mG_2a_ and 47-mG_2a_-f exerted antitumor activity in CHO/hPODXL xenograft models at a dose of 100 μg or 500 μg/mouse/week administered twice. 47-mG_2a_-f, but not 47-mG_2a_, exerted antitumor activity in SAS and HSC-2 xenograft models at a dose of 100 μg/mouse/week administered three times. Although both 47-mG_2a_ and 47-mG_2a_-f exerted antitumor activity in HSC-2 xenograft models at a dose of 500 μg/mouse/week administered twice, 47-mG_2a_-f also showed higher antitumor activity than 47-mG_2a_. These results suggested that a core fucose-deficient anti-PODXL mAb could be useful for antibody-based therapy against PODXL-expressing OSCCs.

## INTRODUCTION

In total, 300,000 cases of oral cancer are reported annually, constituting approximately 2% of all cancer cases globally [1], and several histological tumor types exist, including squamous cell carcinoma (OSCC), adenoid cystic carcinoma, mucoepidermoid carcinoma, and osteosarcoma. Among them, almost 90% of oral cancers are OSCCs [2]. The most common location of OSCC is the tongue, accounting for approximately 40% of OSCCs [3, 4]. Despite improvements in diagnostic technology and therapeutic techniques, the survival rate of OSCC has improved by only 5% over the past 20 years. Consequently, the 5-year survival rate is 60% [5], and the incidence of OSCC has increased globally [4, 6].

OSCC is mainly treated via surgical removal, which can be complemented by radiotherapy and/or chemotherapy, especially in advanced stages. Many types of anticancer agents, including cisplatin (CDDP), 5-fluorouracil (5-FU), and docetaxel, are used for chemotherapy [7, 8]. In contrast, the availability of approved molecular targeting drugs with efficacy against OSCC is limited. Recently, cetuximab, a mouse-human (IgG_1_) chimeric antibody against epidermal growth factor receptor (EGFR), was approved for treating head and neck cancer (HNC) including oral cancer. In several clinical studies, cetuximab was found to be effective against locoregionally advanced head and neck squamous cell carcinoma (HNSCC) or recurrent and/or metastatic (R/M) HNSCC [7, 9–11]. Furthermore, nivolumab, a fully human IgG_4_ mAb against programmed cell death-1, was also approved for the treatment of R/M HNC, which has been treated with platinum-based chemotherapy [12]. In addition, bevacizumab, which is a mouse-human IgG_1_ chimeric antibody against vascular endothelial growth factor (VEGF) and first approved for colorectal cancer, had some clinical trial in which R/M HNSCC patients were enrolled [13]. Because molecular targeting drugs that are clinically applicable for oral cancers are limited, novel drugs with greater efficacy and lower toxicity are required. Therefore, we have been investigating several membrane proteins, and we developed several mAbs against those targets, including HER2 [14], EGFR [15], podoplanin [16], and PODXL [17].

Podocalyxin (PODXL) is a type I transmembrane protein with a molecular weight of 150,000–200,000 [18–20], and it is also known as PCLP, MEP21, Gp200, Gp135, thrombomucin, GCTM2, TRA-1-60 antigen, and TRA-1-81 antigen. PODXL is highly glycosylated with *N*- and *O*-glycans [21]. It can be found in kidney, heart, pancreatic, and breast tissues, and it plays important roles in the development of some tissues [22]. PODXL is also a pluripotent stem cell marker [23–26]. Furthermore, it is a diagnostic marker and a prognostic indicator for certain cancers such as brain tumors [21], colorectal cancer [27], renal cancer [28], and oral cancer [29]. Moreover, PODXL has been suggested to promote tumor growth, invasion, and metastasis [30, 31]; accordingly, high PODXL expression could have adverse effects on overall survival (OS), disease-specific survival (DSS), and disease-free survival (DFS) in several cancers. PODXL is overexpressed in the aforementioned cancers, identifying the protein as a potential target for antibody therapy.

Despite the development of anti-PODXL monoclonal antibodies (mAbs) [32, 33], the efficacy of these treatments against oral cancers remains to be fully elucidated. We previously immunized mice with recombinant PODXL, which was purified from the culture supernatant of LN229/ectodomain-PODXL cells [17]. One clone, PcMab-47 (mouse IgG_1_, kappa), was successfully produced. We further produced chPcMab-47 from PcMab-47 and investigated its antitumor activity against colorectal cancers [34]. In those studies, we injected human NK cells around the tumors to investigate antitumor activity because chPcMab-47 could not induce ADCC activity using mouse NK cells. Although chPcMab-47 significantly reduced tumor development compared with the effects of control human IgG, its antitumor activity might not be sufficient for antibody-based target therapy. We used human NK cells from one donor; therefore, the cause of the low antitumor activity is due to differences in the sources of cells. Because we had to inject human NK cells around the subcutaneous tumors of xenograft models several times, we experienced difficulties in measuring tumor diameters because the tumor shape sometimes changed post-injection. Furthermore, mouse IgG_1_ does not induce ADCC and CDC, and PcMab-47 was determined to be IgG_1_. In this study, we established the 47-mG_2a_, a chimeric anti-PODXL antibody by combining variable region of PcMab-47 and constant region of mouse IgG_2a_. We further produced 47-mG_2a_-f, a core fucose-deficient 47-mG_2a_ to analyze antitumor activity in xenograft models [35].

## RESULTS

### Production of the mouse IgG_2a_-type antibody PcMab-47

**Figure 1:**
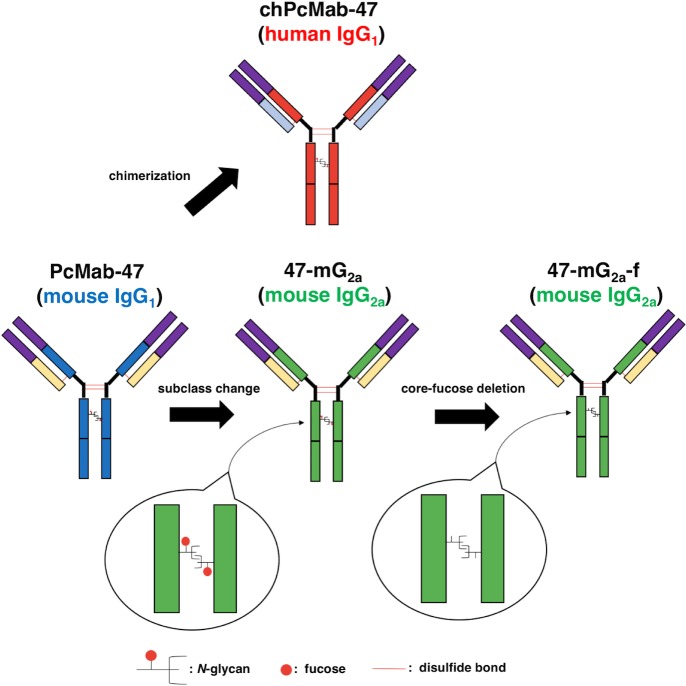
Schematic illustration of PcMab-47, 47-mG_2a_ and 47-mG_2a_-f 47-mG_2a_ was produced by subcloning appropriate V_H_ and V_L_ cDNAs of PcMab-47 and C_H_ and C_L_ of mouse IgG_2a_ into pCAG vectors. 47-mG_2a_-f was further produced by defucosylating 47-mG_2a_.

In this study, we first produced a mouse IgG_2a_-type version of PcMab-47 by subcloning appropriate V_H_ and V_L_ cDNAs of PcMab-47 and C_H_ and C_L_ of mouse IgG_2a_ into pCAG vectors because mouse IgG_2a_ possess high ADCC and CDC activities (Figure 1) [36]. The IgG_2a_-type PcMab-47 was designated 47-mG_2a_.

**Figure 2:**
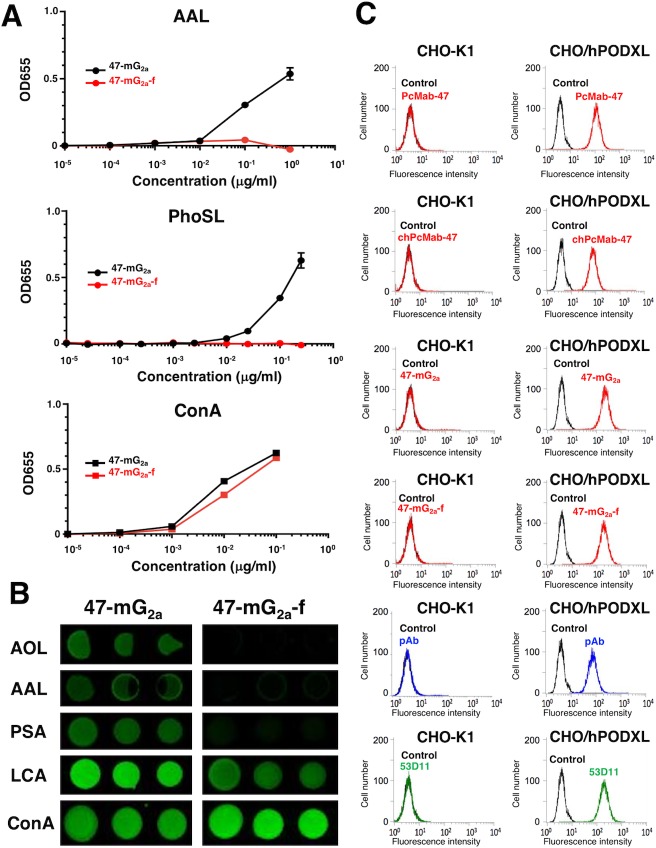
Confirmation of the defucosylation of 47-mG_2a_-f by lectins **(A)** Enzyme-linked immunosorbent assay (ELISA) using lectins. 47-mG_2a_ and 47-mG_2a_-f were immobilized and incubated with biotin-labeled lectins such as *Aleuria aurantia* lectin (AAL), *Pholiota squarrosa* lectin (PhoSL), and concanavalin A (Con A) followed by peroxidase-conjugated streptavidin. The enzymatic reaction was produced using a 1-Step Ultra TMB-ELISA. **(B)** Lectin microarray. AOL, *Aspergillus oryzae* lectin; PSA, *Pisum sativum* agglutinin; LCA, *Lens culinaris* agglutinin. **(C)** Flow cytometry using anti-PODXL antibodies. Cells were treated with PcMab-47 (1 μg/mL), chPcMab-47 (1 μg/mL), 47-mG_2a_ (1 μg/mL), 47-mG_2a_-f (1 μg/mL), polyclonal anti-PODXL antibody (10 μg/mL), or 53D11 (10 μg/mL) followed by secondary antibodies. Black line, negative control. pAb, polyclonal antibody.

We further produced 47-mG_2a_-f (Figure 1). Defucosylation was confirmed using lectins such as *Aleuria aurantia* lectin (AAL, fucose binder) [37] and *Pholiota squarrosa* lectin (PhoSL, core fucose binder) [38]. Concanavalin A (ConA, mannose binder) [39] was used as a control. Both 47-mG_2a_ and 47-mG_2a_-f were detected using ConA (Figure 2A). 47-mG_2a_, but not 47-mG_2a_-f, was detected using AAL and PhoSL (Figure 2A), indicating that 47-mG_2a_-f was defucosylated. We also confirmed the defucosylation using a lectin microarray (Figure 2B). Although 47-mG_2a_ was recognized by core fucose binders such as *Aspergillus oryzae* lectin (AOL) [40], AAL, and *Pisum sativum* agglutinin (PSA) [41], these binders did not detect 47-mG_2a_-f. 47-mG_2a_ was strongly detected using *Lens culinaris* agglutinin (LCA, core fucose and agalactosylated *N*-glycan binder) [42]; in contrast, 47-mG_2a_-f was moderately detected. Both 47-mG_2a_ and 47-mG_2a_-f were detected using ConA. These results indicate that 47-mG_2a_-f was defucosylated. All anti-PODXL antibodies reacted with CHO/hPODXL cells, but not with parental CHO-K1 cells (Figure 2C).

**Figure 3:**
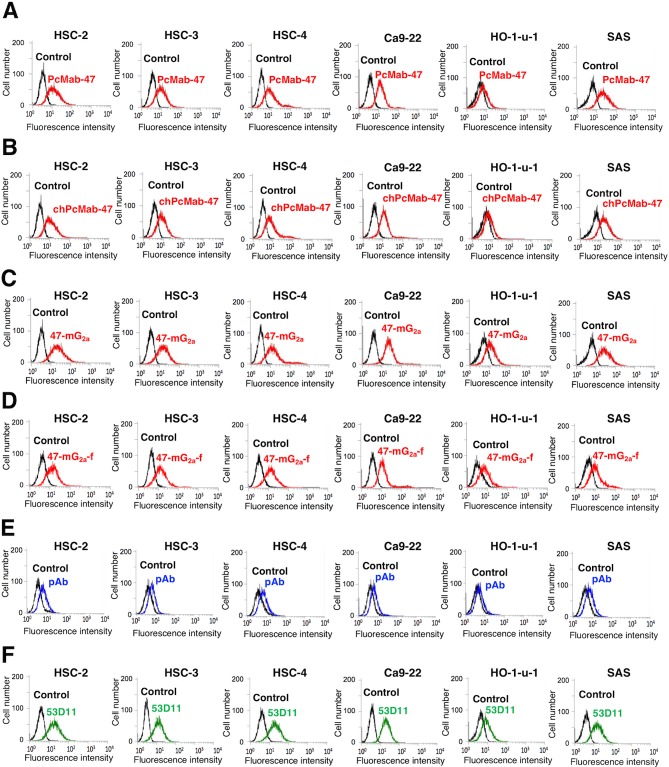
Flow cytometry using anti-PODXL antibodies Cells were treated with PcMab-47 (1 μg/mL) **(A)**, chPcMab-47 (1 μg/mL) **(B)**, 47-mG_2a_ (1 μg/mL) **(C)**, 47-mG_2a_-f (1 μg/mL) **(D)**, polyclonal anti-PODXL antibody (10 μg/mL) **(E)**, or 53D11 (10 μg/mL) **(F)** followed by secondary antibodies. Black line, negative control.

We confirmed the PODXL expression in OSCC cell lines such as HSC-2, HSC-3, HSC-4, Ca9-22, HO-1-u-1, and SAS cells using RT-PCR (data not shown). We examined the sensitivity of 47-mG_2a_ against these OSCC cell lines using flow cytometry. As shown in Figure 3A, IgG_1_-type PcMab-47 recognized endogenous PODXL, which is expressed in OSCC cell lines such as HSC-2, HSC-3, HSC-4, Ca9-22, HO-1-u-1, and SAS cells. PcMab-47 has weaker reactivity against HO-1-u-1 cells than against the other cell lines. The mouse-human chimeric chPcMab-47 reacted with OSCC cells similarly as PcMab-47 (Figure 3B). Furthermore, 47-mG_2a_ and 47-mG_2a_-f exhibited similar reactivity against OSCC cell lines (Figure 3C and 3D). 47-mG_2a_ and 47-mG_2a_-f exhibited greater reactivity against HO-1-u-1 cells, indicating that 47-mG_2a_ and 47-mG_2a_-f are more sensitive for PODXL than PcMab-47. Polyclonal antibody against PODXL reacted with all OSCC cell lines although the reactivity was lower than PcMab-47 (Figure 3E). Another anti-PODXL mAb (clone 53D11) reacted them in the similar pattern with PcMab-47.

### The binding affinity of mouse IgG_2a_-type PcMab-47

**Figure 4:**
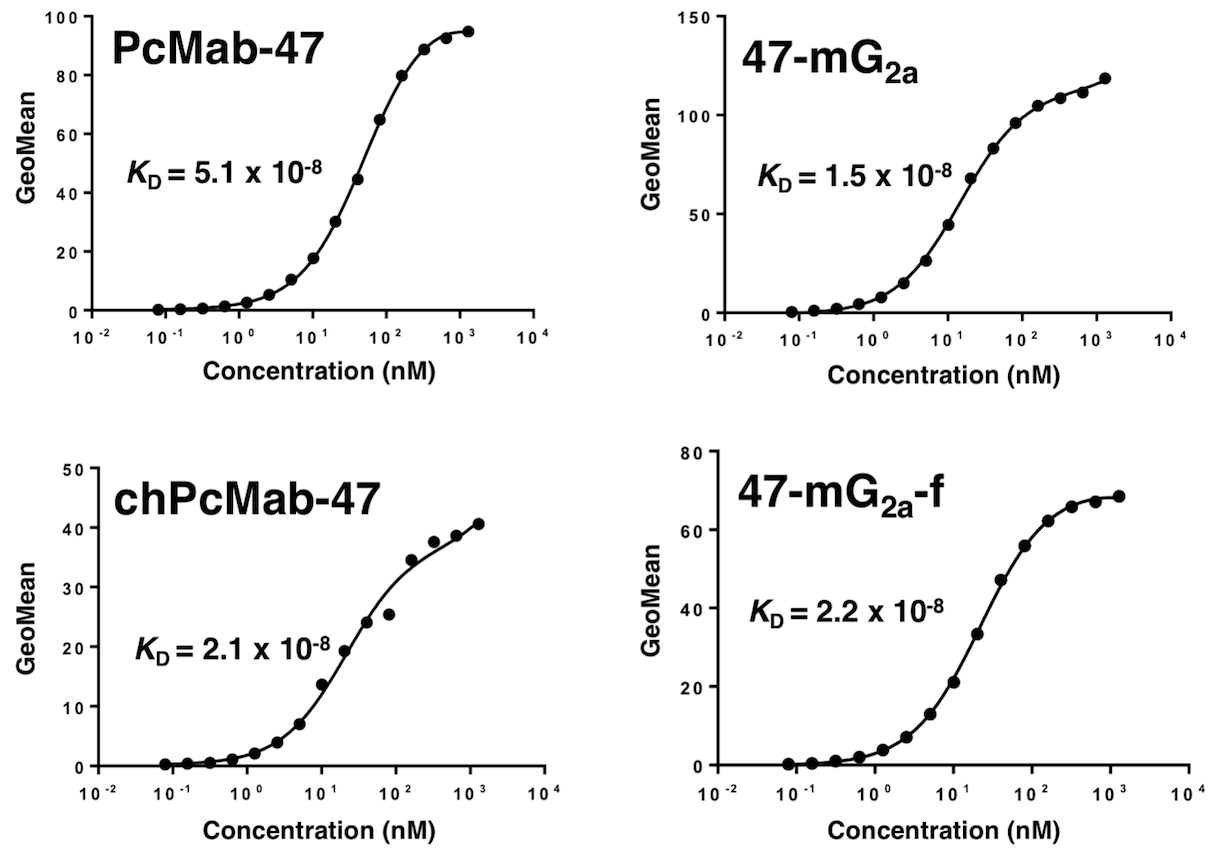
Binding affinities of anti-PODXL antibodies were determined using flow cytometry Ca9-22 cells were suspended in 100 μL of serially diluted antibodies (6 ng/mL to 100 μg/mL), and secondary antibodies were then added. Fluorescence data were collected using a cell analyzer. GeoMean, geometric mean of fluorescence intensity.

We performed a kinetic analysis of the interactions of PcMab-47, chPcMab-47, 47-mG_2a_, and 47-mG_2a_-f with OSCC cells using flow cytometry. As shown in Figure 4, the dissociation constant (*K*_D_) for PcMab-47 against Ca9-22 was 5.1 × 10^-8^ M. In contrast, those for chPcMab-47, 47-mG_2a_, and 47-mG_2a_-f were 2.1 × 10^−8^, 1.5 × 10^−8^, and 2.2 × 10^−8^ M, respectively. The binding affinities of chPcMab-47, 47-mG_2a_, and 47-mG_2a_-f against Ca9-22 were 2.4-, 3.4-, and 2.3-fold higher, respectively, than that of PcMab-47. We further determined the *K*_D_ for PcMab-47 against HSC-2 and SAS cells. The results were summarized in Table 1. The binding affinities of chPcMab-47 and 47-mG_2a_ for HSC-2 cells were 1.1- and 1.5-fold higher than that of PcMab-47, respectively, although 47-mG_2a_-f had lower binding affinity for HSC-2 cells than PcMab-47. The binding affinities of chPcMab-47, 47-mG_2a_, and 47-mG_2a_-f for SAS cells were 1.7-, 1.8-, and 1.1-fold higher than that of PcMab-47, respectively, indicating that all chimeric antibodies, including chPcMab-47, 47-mG_2a_, and 47-mG_2a_-f, possess higher affinity for PODXL than the original PcMab-47. The binding affinities of 47-mG_2a_ for HSC-2, SAS, and Ca9-22 cells were 3.1-, 1.6-, and 1.5-fold higher than those of 47-mG_2a_-f, respectively, indicating that 47-mG_2a_ has greater binding affinity than 47-mG_2a_-f for OSCC cell lines. The difference of binding affinity among cell lines was observed probably because the glycosylation of PODXL might be different in those OSCC cell lines.

**Table 1:** *K*_D_ of anti-PODXL mAbs against OSCC cell lines

	PcMab-47	chPcMab-47	47-mG_2a_	47-mG_2a_-f
HSC-2	2.3 × 10^-8^ M	2.1 × 10^-8^ M	1.5 × 10^-8^ M	4.6 × 10^-8^ M
SAS	8.5 × 10^-9^ M	4.9 × 10^-9^ M	4.6 × 10^-9^ M	7.4 × 10^-9^ M
Ca9-22	5.1 × 10^-8^ M	2.1 × 10^-8^ M	1.5 × 10^-8^ M	2.2 × 10^-8^ M

### Immunohistochemical analysis of PcMab-47, 47-mG_2a_, and 47-mG_2a_-f in OSCC tissues

**Figure 5:**
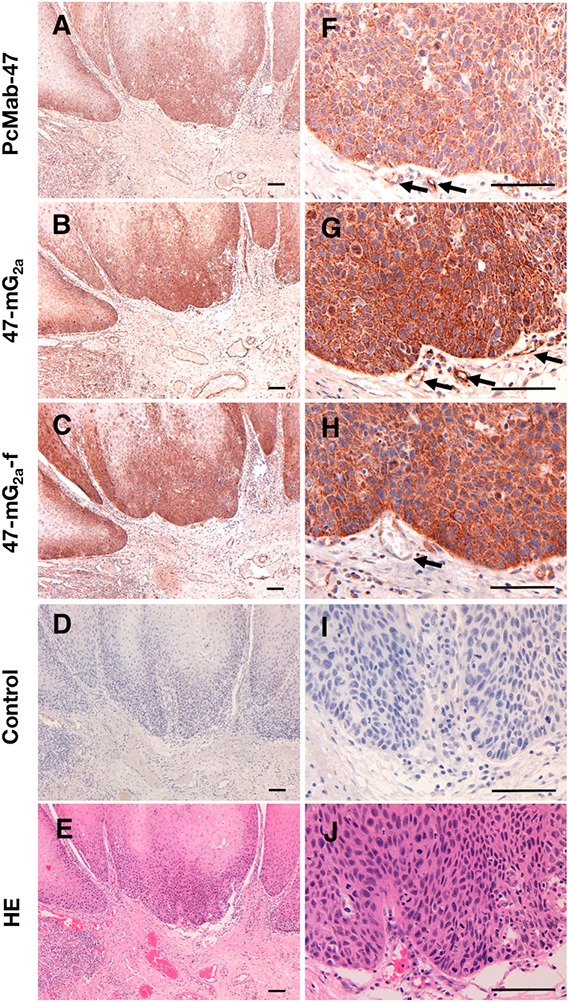
Immunohistochemical analysis of anti-PODXL antibodies in oral squamous cell carcinomas Tissue sections (obtained from Case 174) were incubated with 5 μg/mL PcMab-47 **(A, F)**, 0.5 μg/mL of 47-mG_2a_ **(B, G)**, 0.5 μg/mL 47-mG_2a_-f **(C, H)**, or control (blocking buffer; **D, I**) for 1 h at room temperature followed by treatment with an Envision+ kit for 30 min. Color was developed using 3,3-diaminobenzidine tetrahydrochloride for 2 min, and sections were then counterstained with hematoxylin. **(E, J)** Hematoxylin & eosin staining. Arrows: endothelial cells. Scale bar = 100 μm.

We next performed immunohistochemical analysis in OSCCs using PcMab-47, 47-mG_2a_, and 47-mG_2a_-f. As shown in Figure 5A and 5F, PcMab-47 stained OSCCs in a cytoplasmic staining pattern; contrarily, no signals were observed for the negative control (no primary mAb) in a serial section of OSCCs (Figure 5D and 5I). In addition, 47-mG_2a_ and 47-mG_2a_-f also stained OSCCs sensitively (Figure 5B, 5C, 5G, and 5H). Of interest, the concentration used for 47-mG_2a_ and 47-mG_2a_-f in immunohistochemical analysis was 0.5 μg/mL, which was 10-fold lower than that for PcMab-47, indicating that 47-mG_2a_ and 47-mG_2a_-f are extremely sensitive for use in OSCC tissues. PcMab-47, 47-mG_2a_, and 47-mG_2a_-f also stained endothelial cells around tumor cells (Figure 5F, 5G, and 5H). The sensitivity of 47-mG_2a_ and 47-mG_2a_-f was extremely similar in immunohistochemical analysis, and we used PcMab-47 and 47-mG_2a_ for further immunohistochemical studies.

We stained 201 OSCC samples using PcMab-47 and 47-mG_2a_ and summarized our findings in Table 2. PcMab-47 stained 163/201 (81.1%) OSCC samples. Among them, the intensity of staining was 3+ in 11/163 (6.7%) samples. In contrast, 47-mG_2a_ stained 197/201 (98.0%) OSCC samples. Among them, the intensity was 3+ in 15/197 (7.6%) samples, indicating that 47-mG_2a_ is more sensitive than PcMab-47 in immunohistochemical analyses of OSCCs. All staining results for PcMab-47 and 47-mG_2a_ are shown in Supplementary Table 1.

**Table 2:** Summary of anti-PODXL immunostaining in OSCC

	Score				No. of positive cases	Positive rate (%)
	-	1+	2+	3+		
PcMab-47	38	120	32	11	163/201	81.1
47-mG_2a_	4	134	48	15	197/201	98.0

The intensity of staining was evaluated as -, 1+, 2+, or 3+.

**Figure 6:**
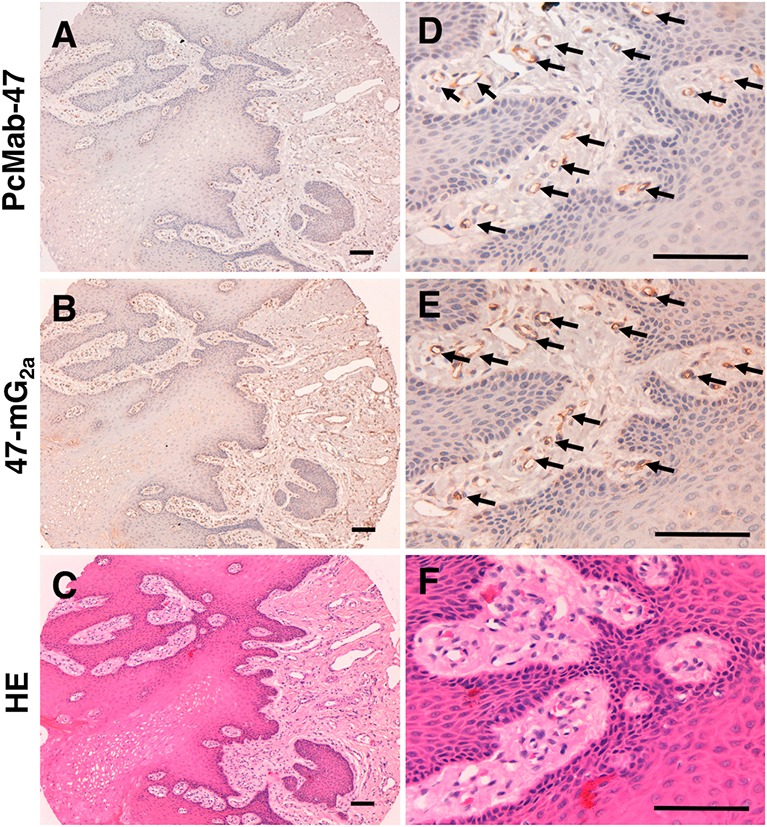
Immunohistochemical analysis of anti-PODXL antibodies in normal tongue Tissue sections of normal tongue were incubated with 5 μg/mL PcMab-47 **(A, D)** and 0.5 μg/mL of 47-mG_2a_ **(B, E)** for 1 h at room temperature followed by treatment with an Envision+ kit for 30 min. Color was developed using 3,3-diaminobenzidine tetrahydrochloride for 2 min. Sections were then counterstained with hematoxylin. **(C, F)** Hematoxylin & eosin staining. Arrows: endothelial cells. Scale bar = 100 μm.

We also performed immunohistochemical analysis using PcMab-47 and 47-mG_2a_ against normal tongue, and checked the PODXL expression in normal squamous epithelium. As shown in Figure 6, PODXL was not detected in normal squamous epithelium. In contrast, PODXL was detected in normal endothelial cells. The staining intensity of 47-mG_2a_ was also higher than that of PcMab-47 in normal endothelial cells. We further checked the cross-reactivity of PcMab-47 against mouse PODXL using flow cytometry and immunohistochemistry. However, no reaction was observed against mouse PODXL (data not shown), indicating that anti-PODXL antibodies might not affect the tumor angiogenesis or tumor microenvironment in mouse xenograft model.

### Functional analysis of PODXL in OSCC cells *in vitro* and *in vivo*

**Figure 7:**
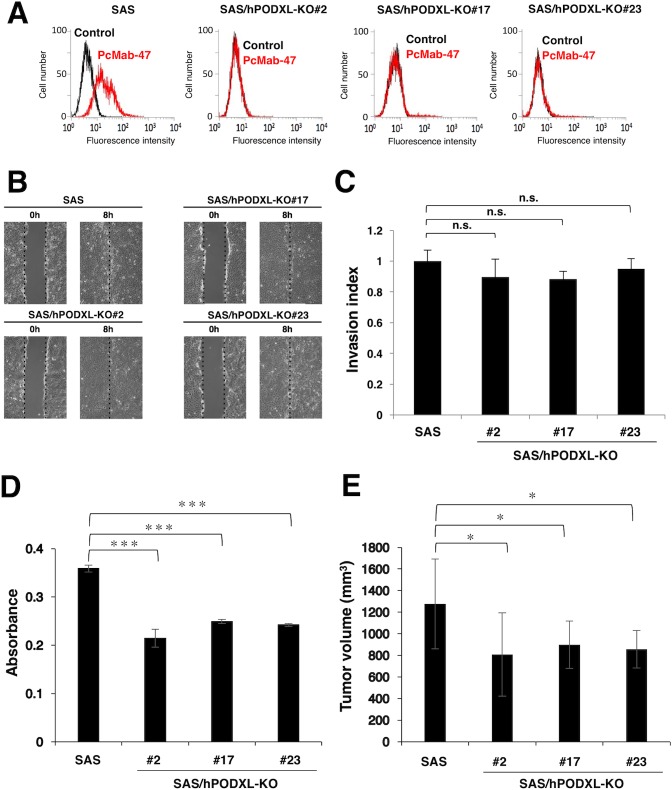
Functional analysis of PODXL *in vitro* and *in vivo* using PODXL-knockout (KO) oral squamous cell carcinoma cell lines **(A)** Flow cytometry. Cells were treated with 10 μg/mL PcMab-47 followed by secondary antibodies. Black line, negative control. **(B)** Evaluation of cell migration. A wound-healing assay was performed to examine the effects of PODXL-KO on SAS cell migration. Images of wounded cell monolayers taken at 0 and 8 h. The vertical lines indicate the wound edge. **(C)** Evaluation of cell invasion using the Transwell invasion assay. The histogram indicates the average of three experiments (values are means ± SEM) and shows the invasion ratio normalized to the value in parental SAS cells. n.s., not significant. **(D)** The MTS assay was performed to determine cell growth. The values are means ± SEM. An asterisk indicates statistical significance between SAS and SAS/hPODXL-KO cells (^***^P < 0.005, two-tailed Student's *t*-test). **(E)** *In vivo* analysis. SAS and SAS/hPODXL-KO cells were injected subcutaneously into female nude mice. The tumor volume was measured at day 21. The values are means ± SEM. An asterisk indicates statistical significance between SAS and SAS/hPODXL-KO cells (^*^P < 0.05, two-tailed Student's *t*-test).

Next, we investigated whether PODXL is associated with tumor phenotype in OSCC cell lines. We selected SAS cells for this study because they express PODXL at higher levels than other OSCC cells (Figure 3) and they have been reported to grow extremely well *in vivo* [43]. As shown in Figure 7A, PcMab-47 did not react with PODXL-knockout (KO) SAS cells (SAS/hPODXL-KO). To examine the migratory and invasive abilities of SAS/hPODXL-KO cells, we performed wound-healing and invasion assays, respectively, but no significant differences in migration (Figure 7B) and invasion (Figure 7C) were identified between parental and SAS/hPODXL-KO cells. We next investigated whether PODXL is associated with the growth of OSCC cell lines *in vitro* using the MTS assay. The growth of three SAS/hPODXL-KO cell lines was lower than that of parental SAS cells (Figure 7D). We further investigated whether PODXL affects OSCC tumor growth *in vivo* by comparing the growth of SAS and three SAS/hPODXL-KO cell lines that were transplanted subcutaneously into nude mice. As shown in Figure 7E, the growth of SAS/hPODXL-KO cells was lower than that of parental SAS cells.

**Figure 8:**
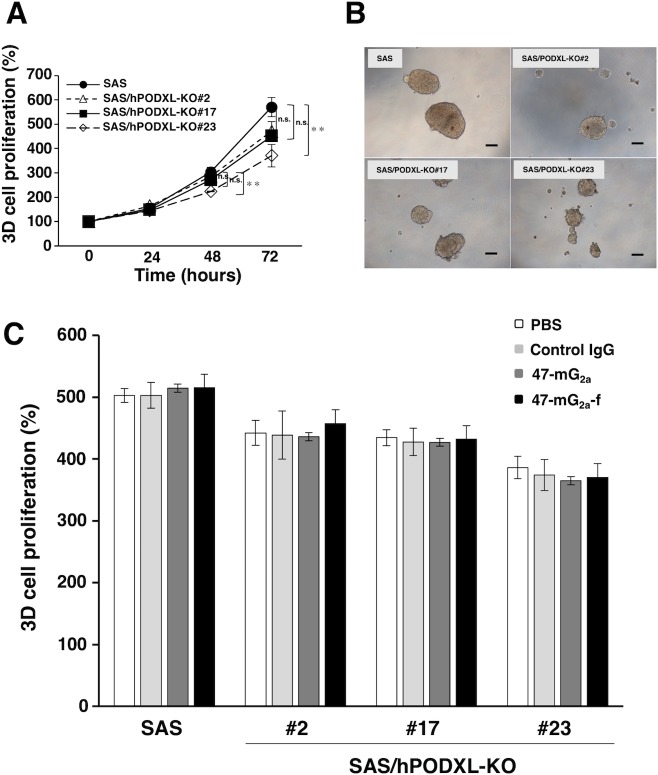
Proliferation of SAS and SAS/hPODXL-KO cells on 3D cultures and the influence of anti-PODXL antibodies on the 3D cell proliferation **(A)** 3D cell proliferation was measured by 3D cell proliferation assay. The graphs show the relative cell growth in 3D culture at 24, 48, and 72 h. Each bar represents the mean ± SEM. An asterisk indicates statistical significance (^**^P < 0.01, Tukey-Kramer's test). **(B)** Optical microscopic images of SAS and SAS/hPODXL-KO cells after 72 h incubation. Scale bar, 200 μm. **(C)** 3D cell proliferation treated with or without anti-hPODXL antibodies (47-mG_2a_ and 47-mG_2a_-f) for 72 h. The values are means ± SEM.

Furthermore, we investigated the effect of PODXL on the growth of 3D cells with cancer stem cell-like properties. The 3D growth of three SAS/hPODXL-KO cell lines tended to decrease, and the growth of SAS/hPODXL-KO#23 cells was significantly inhibited after incubation for 72 h, indicating that PODXL has tumorigenic potential and might be associated with tumorigenicity *in vivo* (Figure 8A and 8B). In contrast, there are little effects by anti-PODXL antibodies on 3D cell proliferation *in vitro* (Figure 8C).

### ADCC and CDC activities of 47-mG_2a_ and 47-mG_2a_-f

**Figure 9:**
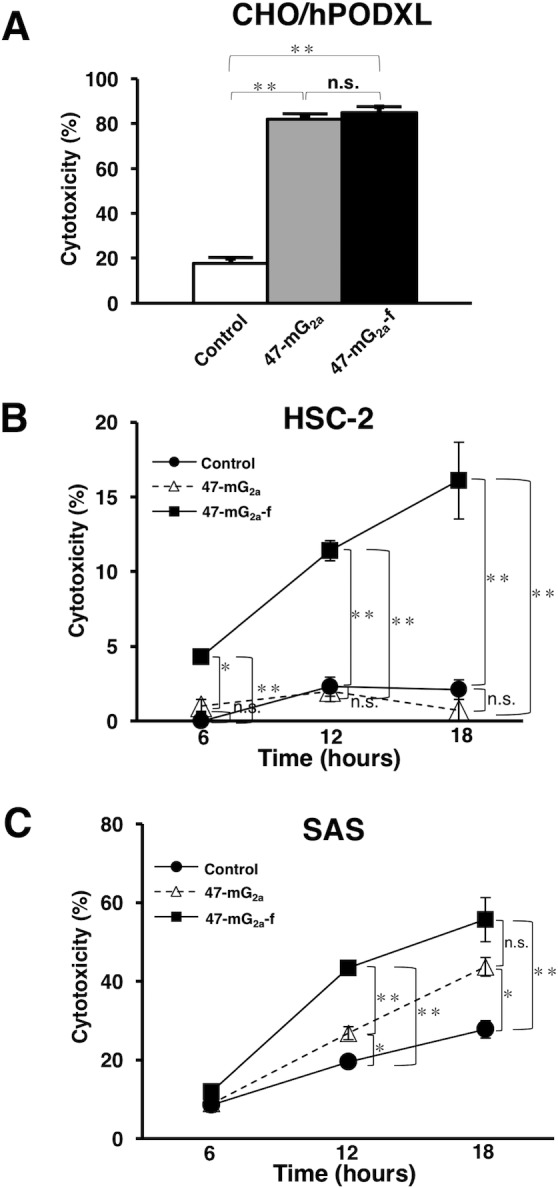
Complement-dependent cytotoxicity (CDC) and antibody-dependent cellular cytotoxicity (ADCC) of anti-PODXL mAbs against PODXL-expressing cell lines **(A)** CDC was evaluated using the ^51^Cr release assay. Target cells were incubated with [^51^Cr]sodium chromate (0.1 μCi) for 1 h at 37°C. The ^51^Cr-labeled cells were incubated with a baby rabbit complement at a dilution of 1:4 in the presence of 47-mG_2a_, 47-mG_2a_-f, or control mouse IgG (10 μg/mL) for 6 h in 96-well plates. ^51^Cr release was measured in the supernatant. **(B, C)** ADCC was determined using the ^51^Cr release assay. Target cells such as HSC-2 (B) and SAS (C) were incubated with [^51^Cr]sodium chromate. After washing, ^51^Cr-labeled target cells were placed in 96-well plates in triplicate. Effector cells and 47-mG_2a_, 47-mG_2a_-f, or control mouse IgG were added to the plates. After incubation (6, 12, and 18 hrs), ^51^Cr release was measured in the supernatant. The values are means ± SEM. ^*^P < 0.05, ^**^P < 0.01 *t*-test.

We investigated whether 47-mG_2a_ and 47-mG_2a_-f can induce ADCC and CDC in PODXL-expressing cell lines. Both 47-mG_2a_ and 47-mG_2a_-f induced CDC against CHO/hPODXL (Figure 9A), whereas they showed faint ADCC against CHO/hPODXL cells (data not shown), indicating that only CDC is critical for the antitumor activity of 47-mG_2a_-f against CHO/hPODXL cells.

47-mG_2a_-f induced ADCC activity against HSC-2, whereas 47-mG_2a_ did not (Figure 9B). Furthermore, higher ADCC was observed by 47-mG_2a_-f against SAS cells compared with 47-mG_2a_ (Figure 9C). These results indicate that depletion of core-fucose is important for inducing ADCC against OSCC cells. Neither 47-mG_2a_ nor 47-mG_2a_-f induced CDC in OSCC cell lines (data not shown), indicating that only ADCC is critical for the antitumor activity of 47-mG_2a_-f against OSCC cells.

### Antitumor activity of 47-mG_2a_ and 47-mG_2a_-f against OSCC xenografts

**Figure 10:**
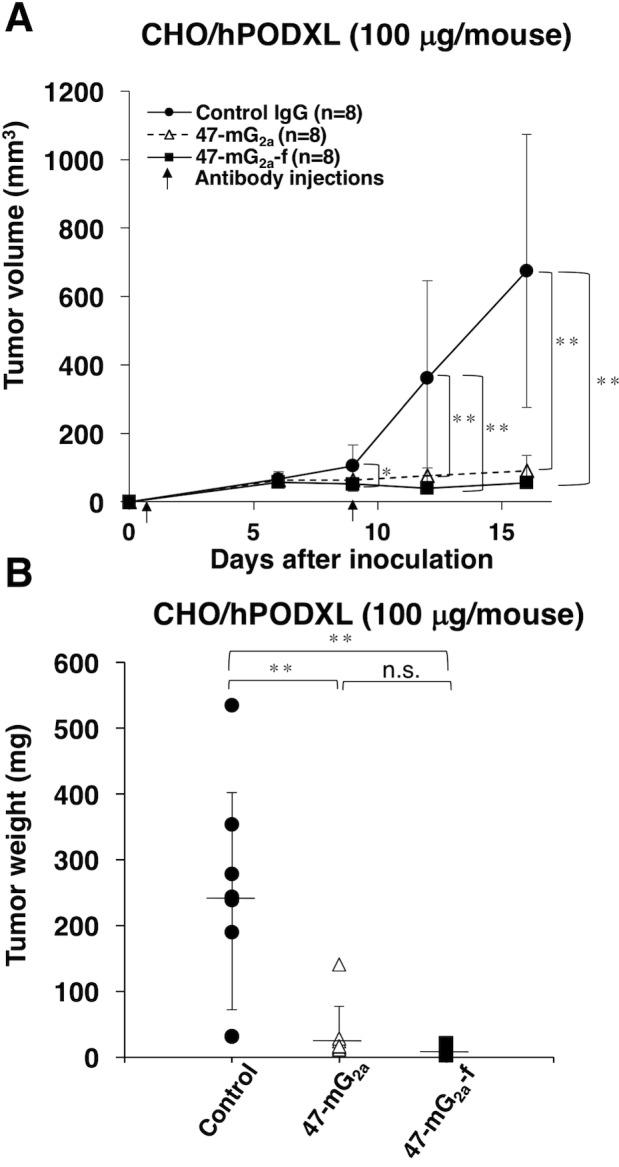
Antitumor activity of 47-mG_2a_ and 47-mG_2a_-f against CHO/hPODXL **(A)** Tumor volume of CHO/hPODXL xenografts. CHO/hPODXL cells were injected subcutaneously into female nude mice. The indicated antibodies (100 μg/day; 5 mg/kg) were administered intraperitoneally 1 and 9 days after cancer cell inoculation. The tumor volume was measured at the indicated time points. The values are means ± SEM. **(B)** Tumor weight of CHO/hPODXL xenografts (day 16). The values are means ± SEM. An asterisk indicates statistical significance (^*^P < 0.05, ^**^P < 0.01, Tukey-Kramer's test).

To study the antitumor effects of 47-mG_2a_ and 47-mG_2a_-f on primary tumor growth *in vivo*, CHO/hPODXL cells were subcutaneously implanted into the flanks of nude mice. 47-mG_2a_ and 47-mG_2a_-f, and mouse IgG (control) were injected twice (100 μg of the antibodies on days 1 and 9 after cell inoculation) into the peritoneal cavity of mice. Tumor formation was observed in all groups. Both 47-mG_2a_ and 47-mG_2a_-f significantly reduced tumor development compared with IgG on day 16 (Figure 10A). The tumor weights of mice in the both 47-mG_2a_ and 47-mG_2a_-f groups were significantly lower than that in the IgG group (Figure 10B). Body weight was not significantly different among the three groups (Supplementary Figure 1A and 1B). The resected tumors of CHO/hPODXL xenografts are depicted in Supplementary Figure 1C. No difference was illustrated among those groups using hematoxylin & eosin (HE) staining (Supplementary Figure 2). Both 47-mG_2a_ and 47-mG_2a_-f showed the similar results in CHO/hPODXL xenograft models because those mAbs might exert CDC activities against CHO/hPODXL, and might not exert ADCC activities *in vivo*.

**Figure 11:**
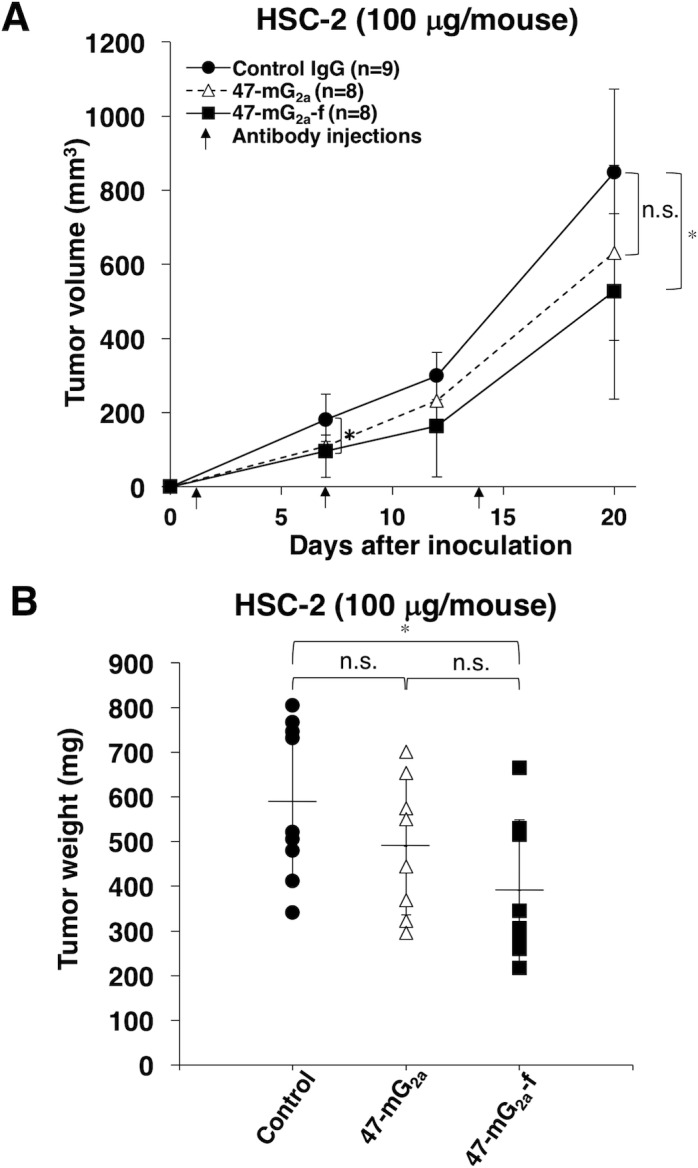
Antitumor activity of 47-mG_2a_ and 47-mG_2a_-f against HSC-2 xenografts **(A)** Tumor volume of HSC-2 xenografts. HSC-2 cells were injected subcutaneously into female nude mice. The indicated antibodies (100 μg/day; 5 mg/kg) were administered intraperitoneally 1, 7, and 14 days after cancer cell inoculation. The tumor volume was measured at the indicated time points. The values are means ± SEM. **(B)** Tumor weight of HSC-2 xenografts (day 20). An asterisk indicates statistical significance (^*^P < 0.05, Tukey-Kramer's test).

We also investigated the antitumor activity of 47-mG_2a_ and 47-mG_2a_-f using HSC-2 xenograft models. 47-mG_2a_ and 47-mG_2a_-f, and mouse IgG (control) were injected three times (100 μg of the antibodies on days 1, 7, and 14 after cell injections) into the peritoneal cavity of mice. Tumor formation was observed in all groups. 47-mG_2a_-f significantly reduced tumor development compared with IgG on day 20 (Figure 11A). Although 47-mG_2a_ also reduced tumor development compared with IgG, the result was not significant, indicating that the depletion of core fucose is critical for the antitumor activity of 47-mG_2a_-f against HSC-2 xenograft models. The tumor weight of mice in the 47-mG_2a_-f group was significantly lower than that in the IgG group (Figure 11B). Conversely, 47-mG_2a_ was not associated with a significant decrease in tumor weight versus IgG. However, body weight was not significantly different among the three groups (Supplementary Figure 3A and 3B). The resected tumors of HSC-2 xenografts are depicted in Supplementary Figure 3C. No difference was illustrated among those groups using HE staining (Supplementary Figure 4).

**Figure 12:**
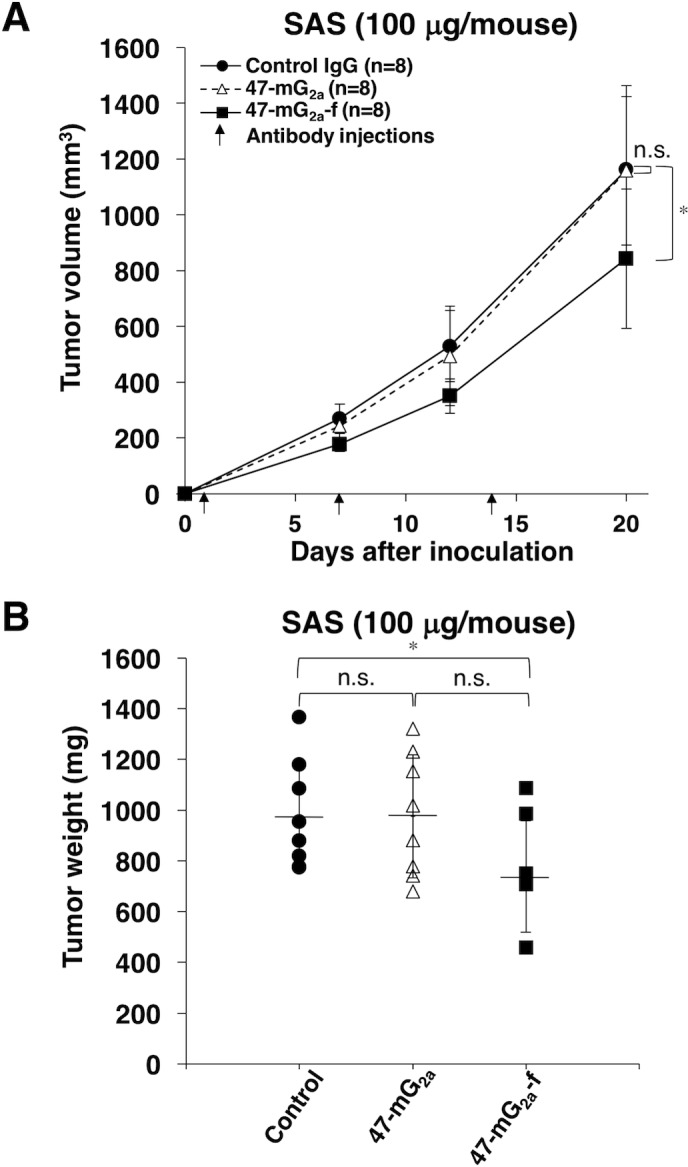
Antitumor activity of 47-mG_2a_ and 47-mG_2a_-f against SAS xenografts **(A)** Tumor volume of SAS xenografts. SAS cells were injected subcutaneously into female nude mice. The indicated antibodies (100 μg/day; 5 mg/kg) were administered intraperitoneally 1, 7, and 14 days after cancer cell inoculation. The tumor volume was measured at the indicated time points. The values are means ± SEM. **(B)** Tumor weight of SAS xenografts (day 20). An asterisk indicates statistical significance (^*^P < 0.05, Tukey-Kramer's test).

We further investigated the antitumor activity of 47-mG_2a_ and 47-mG_2a_-f using SAS xenograft models. In this study, we injected 100 μg of the antibodies three times. As shown in Figure 12A, 47-mG_2a_-f, but not 47-mG_2a_, significantly reduced tumor development compared with IgG on day 20, indicating that the depletion of core fucose is also critical for antitumor activity of 47-mG_2a_-f against SAS xenograft models. Tumor weight was significantly lower in the 47-mG_2a_-f group than in the IgG group (Figure 12B). Conversely, 47-mG_2a_ was not linked to decreases in tumor weight. Body weight was similar among the three groups (Supplementary Figure 5A and 5B). The resected tumors of SAS xenografts are shown in Supplementary Figure 5C. No difference in HE staining was identified among the groups (Supplementary Figure 6).

We performed dose-escalation studies using CHO/hPODXL xenograft models (Supplementary Figure 7). In this study, we injected 500 μg of the antibodies twice. As shown in Supplementary Figure 7A, both 47-mG_2a_ and 47-mG_2a_-f significantly reduced tumor development compared with IgG on day 16 (*P* < 0.01). The resected tumors of CHO/hPODXL xenografts are shown in Supplementary Figure 7B. Tumor weights were significantly lower in the 47-mG_2a_ and 47-mG_2a_-f groups than in the IgG group (Supplementary Figure 7C). Body weight was similar among the three groups (data not shown), as were HE staining patterns (Supplementary Figure 7D). In CHO/hPODXL xenograft models, dose-escalation was not necessary because 100 μg of the antibodies twice showed enough anti-tumor activities.

**Figure 13:**
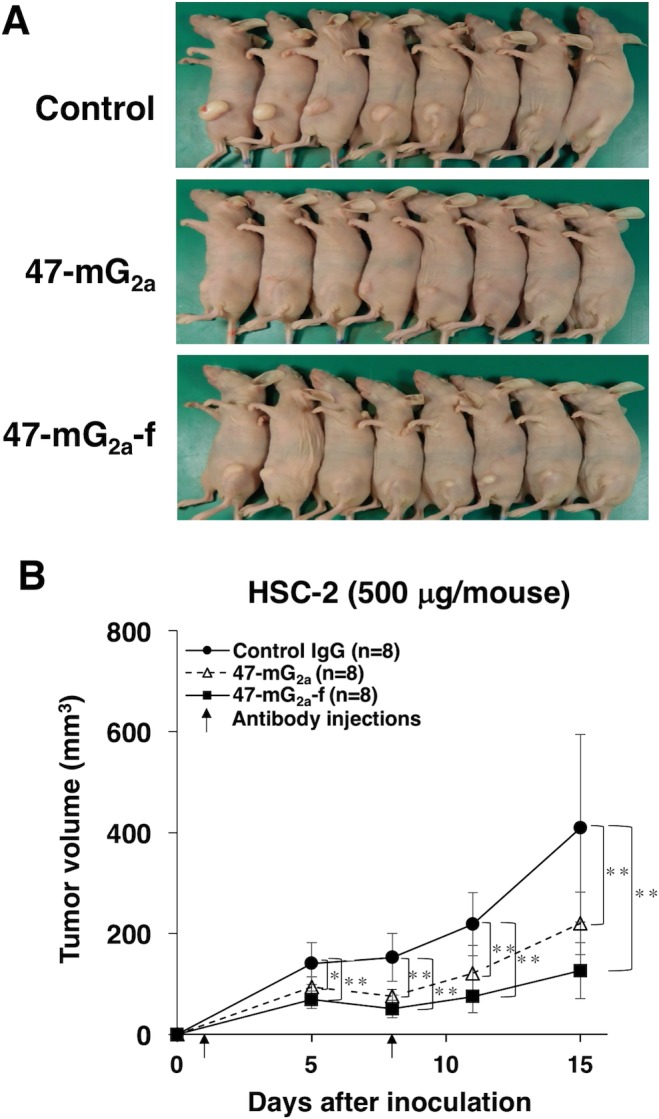
Antitumor activity of 47-mG_2a_ and 47-mG_2a_-f against HSC-2 xenografts (500 μg/day; 25 mg/kg) **(A)** Comparison of the tumor size and tumor incidence of HSC-2 xenograft in nude mice (day 15). **(B)** Tumor volume of HSC-2 xenografts. HSC-2 cells were injected subcutaneously into female nude mice. The indicated antibodies (500 μg/day; 25 mg/kg) were administered intraperitoneally 1 and 7 days after cancer cell inoculation. The tumor volume was measured at the indicated time points. The values are means ± SEM. An asterisk indicates statistical significance (^**^P < 0.01, Tukey-Kramer's test).

**Figure 14:**
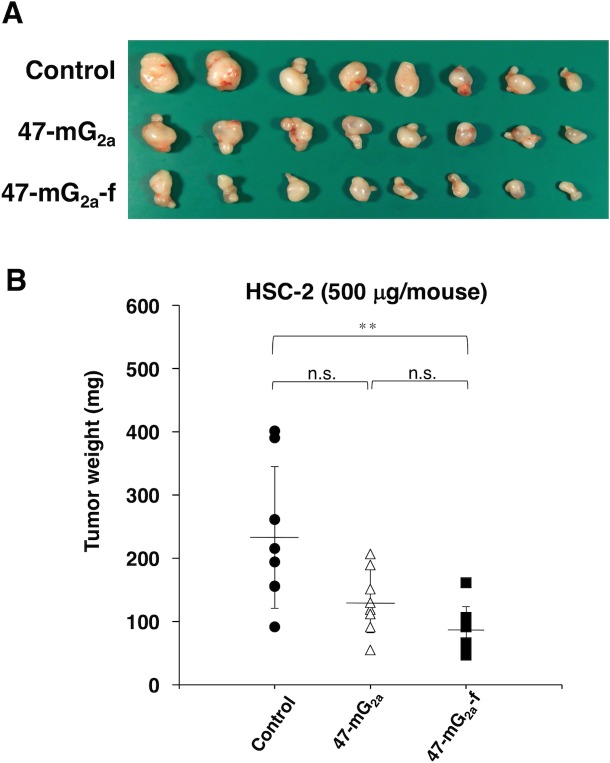
Antitumor activity of 47-mG_2a_ and 47-mG_2a_-f against HSC-2 xenografts (500 μg/day; 25 mg/kg) **(A)** Comparison of tumor size (day 15). **(B)** Tumor weight of HSC-2 xenografts (day 15). An asterisk indicates statistical significance (^**^P < 0.01, Tukey-Kramer's test).

We further performed dose-escalation studies using HSC-2 xenograft models because we could not observe sufficient antitumor activity after three injections of 100 μg of the antibodies. In this study, we injected 500 μg of the antibodies twice. As shown in Figure 13A and 13B, both 47-mG_2a_ and 47-mG_2a_-f reduced tumor development compared with control mouse IgG on day 15 (*P* < 0.01). Furthermore, 47-mG_2a_-f exhibited greater antitumor activity than 47-mG_2a_, but it is not significant (Figure 13B). The resected tumors of HSC-2 xenografts are shown in Figure 14A. Tumor weights were significantly lower in the both 47-mG_2a_ and 47-mG_2a_-f group than in the IgG group (Figure 14B). Body weight was similar among the three groups (data not shown), as were HE staining patterns (Supplementary Figure 8). These results indicate that 47-mG_2a_-f showed higher anti-tumor activities against HSC-2 due to its higher ADCC against PODXL-expressing OSCCs, and dose-escalation was effective for those anti-tumor activities in HSC-2 xenograft models.

We finally performed dose-escalation studies using SAS xenograft models because we could not observe sufficient antitumor activity after three injections of 100 μg of the antibodies (Supplementary Figure 9). In this study, we injected 500 μg of the antibodies three times. As shown in Supplementary Figure 9A, 47-mG_2a_-f reduced tumor development compared with control mouse IgG on day 21 (*P* < 0.05) and exhibited significantly greater antitumor activity than 47-mG_2a_. The resected tumors of SAS xenografts are shown in Supplementary Figure 9B. Tumor weight was significantly lower in the 47-mG_2a_-f group than in the IgG group, whereas 47-mG_2a_ did not reduce tumor weight (Supplementary Figure 9C). Body weight was similar among the three groups (data not shown), as were HE staining patterns (Supplementary Figure 9D). These results indicate that 47-mG_2a_-f showed higher anti-tumor activities due to its higher ADCC against PODXL-expressing OSCCs, but dose-escalation was not sufficient for those anti-tumor activities *in vivo*.

## DISCUSSION

47-mG_2a_ and 47-mG_2a_-f showed higher binding activity compared with its original PcMab-47 (Figure 4). We have sometimes experienced that chimeric antibodies possesses much higher affinity or lower affinity compared with original monoclonal antibodies [34, 44–49]. The stability of antibodies might be different among constant regions. Although 47-mG_2a_ exhibited ADCC activity against SAS cells (Figure 9C), this activity is not sufficient to induce antitumor activity *in vivo* (Figure 12). Then, we produced a non-fucosylated version of 47-mG_2a_ (47-mG_2a_-f) to augment its ADCC activities because non-fucosylated antibodies are known to show higher ADCC activities [35, 50]. As expected, 47-mG_2a_-f exhibited stronger ADCC activities than 47-mG_2a_ against OSCC cells (Figure 9B and 9C), leading to higher antitumor activities in HSC-2 and SAS xenograft models (Figure 11 and 12). In contrast, three injections were not effective despite a dose of 5 mg/kg, which corresponded to 100 μg/mouse in this study and exceeded the usual dose of antibody therapy [51, 52]. Then, two injections of 500 μg/mouse, corresponding to 25 mg/kg, resulted in greater antitumor activities in HSC-2 xenograft models (Figure 13), indicating that dose escalation might be necessary for monotherapy using anti-PODXL antibodies although dose-escalation was not sufficient for SAS xenograft models (Supplementary Figure 9). Furthermore, we need to combine anti-PODXL antibodies with anti-cancer drugs or include them in novel antitumor regimens, including T cells and viruses, to exert antitumor activity against cancer cells. Previously, we produced chPcMab-47, a mouse-human chimeric antibody, which could be applied to cancer patients [34]. chPcMab-47 exerted ADCC and CDC activity and showed antitumor activities in mouse xenograft models. Because 47-mG_2a_-f showed much higher ADCC activity in this study, we also need to produce core-fucose-deficient chPcMab-47 in the future study.

Jing *et al.* reported that high PODXL expression was significantly associated with worse OS and was predictive of shorter OS in multiple cancers, especially pancreatic and colorectal cancers [53]. They also revealed that high PODXL expression predicted worse DSS and DFS, although European patients were included in this analysis. These results suggest PODXL could be a prognostic factor, and diagnostic tools targeting this protein are expected. Because the association between PODXL expression and clinical stage has not been investigated in OSCC, it was analyzed in the current study. However, PODXL expression in stages III–IV was not significantly higher than that in stages I–II in patients with OSCC (data not shown). Further investigation regarding the clinical importance of PODXL expression in more patients with OSCC is needed.

Taken together, anti-PODXL antibodies could be useful antibody therapies against PODXL-expressing OSCCs. PODXL is known to be expressed in kidney, heart, pancreas, and breast tissues, and it plays important roles in those tissues [22]. Recently, we successfully produced cancer-specific mAbs (CasMabs) against human podoplanin [16, 54]. Using the same methods, we need to develop CasMabs against PODXL in the near future.

## MATERIALS AND METHODS

### Cell lines

HSC-2 (oral squamous carcinoma from oral cavity), HSC-3 (oral squamous carcinoma cell line from tongue with high metastatic potential), HSC-4 (oral squamous carcinoma cell line from tongue), Ca9-22 (oral squamous carcinoma from gingiva), HO-1-u-1 (oral squamous carcinoma from mouth floor), and SAS (oral squamous carcinoma cell line from tongue) were obtained from the Japanese Collection of Research Bioresources Cell Bank (Osaka, Japan). CHO-K1 was obtained from the American Type Culture Collection (ATCC, Manassas, VA). CHO/hPODXL was produced in our previous study [17]. The CHO-S cell line was purchased from Thermo Fisher Scientific Inc. (Waltham, MA, USA). PDIS-5 (core fucose KO CHO-S) cells were established previously [48]. SAS/hPODXL-KO cells were produced using CRISPR/Cas9 plasmids (Target ID: HS0000056763) targeting human PODXL (Sigma-Aldrich Corp., St. Louis, MO, USA). HSC-2, HSC-3, HSC-4, Ca9-22, HO-1-u-1, SAS and SAS/hPODXL-KO cells were cultured in Dulbecco's Modified Eagle's Medium (DMEM; Nacalai Tesque, Kyoto, Japan), and CHO-K1 and CHO/hPODXL were cultured in RPMI 1640 medium (Nacalai Tesque), supplemented with 10% heat-inactivated fetal bovine serum (Thermo Fisher Scientific Inc.), 100 units/mL penicillin, 100 μg/mL streptomycin, and 25 μg/mL amphotericin B (Nacalai Tesque) at 37°C in a humidified atmosphere containing 5% CO_2_ and 95% air.

### Tissues

Cancer tissue microarrays of oral cancers were purchased from US Biomax (Rockville, MD, USA) and Cybrdi (Frederick, MD, USA). This study examined 53 patients with oral cancer who underwent surgery at Tokyo Medical and Dental University. The Tokyo Medical and Dental University Institutional Review Board reviewed and approved the use of human cancer tissues. Written informed consent was obtained for the use of human cancer tissue samples.

### Antibodies

PcMab-47, a mouse anti-PODXL mAb (IgG_1_, kappa), was developed as previously described [17]. Mouse IgG was purchased from Sigma-Aldrich. To generate 47-mG_2a_, appropriate V_H_ and V_L_ cDNAs of mouse PcMab-47 and C_H_ and C_L_ of mouse IgG_2a_ were subcloned into pCAG-Ble and pCAG-Neo vectors (Wako Pure Chemical Industries, Osaka, Japan), respectively. Antibody expression vectors were transfected into ExpiCHO-S cells using the ExpiCHO Expression System (Thermo Fisher Scientific). To generate 47-mG_2a_-f, antibody expression vectors were also transfected into PDIS-5 cells using the ExpiCHO Expression System. Stable transfectants of chPcMab-47 cells were established previously [34]. CHO-S/chPcMab-47 cells were cultivated in CHO-S-SFM II medium (Thermo Fisher Scientific). PcMab-47, 47-mG_2a_, 47-mG_2a_-f, and chPcMab-47 were purified using Protein G-Sepharose (GE Healthcare Bio-Sciences, Pittsburgh, PA, USA). Goat polyclonal anti-human PODXL and mouse monoclonal anti-human PODXL mAb (53D11) were purchased from R&D systems, Inc. (Minneapolis, MN) and Medical & Biological Laboratories Co., Ltd. (MBL; Nagoya, Japan), respectively.

### Enzyme-linked immunosorbent assay (ELISA)

47-mG_2a_ and 47-mG_2a_-f were immobilized on Nunc Maxisorp 96-well immunoplates (Thermo Fisher Scientific) at 1 μg/mL for 30 min. After blocking using SuperBlock buffer (Thermo Fisher Scientific) with 0.5 mM CaCl_2_, the plates were incubated with biotin-labeled lectins, such as AAL (Vector Laboratories, Burlingame, CA, USA), PhoSL (J-OIL MILLS, Inc., Tokyo, Japan) [38], and ConA (Vector Laboratories), followed by 1:3000 diluted peroxidase-conjugated streptavidin (Agilent Technologies, Santa Clara, CA, USA). The enzymatic reaction was produced using a 1-Step Ultra TMB-ELISA (Thermo Fisher Scientific). The optical density was measured at 655 nm using an iMark microplate reader (Bio-Rad Laboratories, Inc., Berkeley, CA).

### Lectin microarray

47-mG_2a_ and 47-mG_2a_-f (100 μL; 31.25–2000 ng/mL) were applied to a lectin array (LecChip ver. 1.0; GlycoTechnica, Hokkaido, Japan), including triplicate spots of 45 lectins in each of seven incubation baths on the glass slide. After incubation at 20°C for 17 h, the reaction solution was discarded. The glass slide was scanned using a GlycoStation Reader 1200 (GlycoTechnica).

### Flow cytometry

Cell lines were harvested via a brief exposure to 0.25% trypsin/1 mM ethylenediaminetetraacetic acid (Nacalai Tesque). After washing with 0.1% bovine-serum albumin in phosphate-buffered saline (Nacalai Tesque), cells were treated with primary mAbs for 30 min at 4°C, followed by treatment with Alexa Fluor 488-conjugated anti-mouse IgG (1:1000; Cell Signaling Technology, Danvers, MA, USA), FITC-labeled goat anti-human IgG (1:1000; Thermo Fisher Scientific), or FITC-labeled rabbit anti-goat IgG (1:1000; Thermo Fisher Scientific). Fluorescence data were collected using an EC800 Cell Analyzer (Sony Corp., Tokyo, Japan).

### Determination of binding affinity using flow cytometry

HSC-2, SAS, and Ca9-22 cells (2 × 10^5^) were resuspended in 100 μL of serially diluted PcMab-47, 47-mG_2a_, 47-mG_2a_-f, or chPcMab-47 (6 ng/mL to 100 μg/mL), followed by the addition of secondary anti-mouse IgG (Cell Signaling Technology) or anti-human IgG (Thermo Fisher Scientific). Fluorescence data were collected using a cell analyzer (EC800). *K*_D_ was obtained by fitting the binding isotherms using the built-in one-site binding models in GraphPad PRISM 6 (GraphPad Software, La Jolla, CA, USA).

### Immunohistochemical analyses

Histologic sections (4 μm thick) were deparaffinized in xylene and then rehydrated and autoclaved in citrate buffer (pH 6.0; Agilent Technologies Inc.) for 20 min. Sections were then incubated with 0.5–5 μg/mL primary mAbs for 1 h at room temperature and then treated using an Envision+ kit (Agilent Technologies) for 30 min. Color was developed using 3,3-diaminobenzidine tetrahydrochloride (Agilent Technologies) for 2 min, and sections were then counterstained with hematoxylin (Wako Pure Chemical Industries Ltd.). The intensity of staining was evaluated as −, 1+, 2+, or 3+.

### Proliferation assay *in vitro*

Cell proliferation *in vitro* was measured using MTS tetrazolium (Cell Titer 96 Aqueous, Promega, Madison, WI, USA). Cells were plated (500 cells/100 μL/well) in triplicate in 96-well plates. Cell viability was measured every 24 h for 96 h. After adding 20 μL of MTS to the wells followed by a 1-h incubation at 37°C, the absorbance at 490 nm (reference, 630 nm) was read using a microplate reader (Power Scan HT, Bio Tek Instruments, Winooski, VT, USA). The mean absorbance of the 3-well set was obtained from 0 to 96 h. Statistical significance was analyzed using the standard Student's *t*-test. *P*-values <0.05 were considered statistically significant.

### Proliferation assay *in vivo*

Five-week-old female BALB/c nude mice were purchased from Charles River (Kanagawa, Japan). Seven-week-old mice were used for the *in vivo* proliferation assay. Cells (0.3 mL of 1.33 × 10^8^ /mL in DMEM) were mixed with 0.5 mL of BD Matrigel Matrix Growth Factor Reduced (BD Biosciences, San Jose, CA, USA). A 100 μL suspension (containing 5 × 10^6^ cells) was injected subcutaneously into the left flanks of nude mice. The tumor diameter was measured using calipers, and the tumor volume was calculated using the following formula: volume = W^2^ × L/2, where W is the short diameter and L is the long diameter. The mice were euthanized 21 days after cell implantation. All data were expressed as the mean ± SEM. Statistical analysis was performed using a two-tailed Student's *t*-test. *P*-values <0.05 were considered statistically significant.

### Invasion assay

The invasion assay was performed using a Cytoselect 96-well Collagen Cell Invasion Assay Kit (Cell Biolabs, San Diego, CA, USA) according to the manufacturer's instructions. Cells (2.5 × 10^4^) were pre-incubated in DMEM containing 0.5% FBS for 12 h, washed with serum-free DMEM, and added to the top insert of a chamber containing a polycarbonate membrane with 8-μm pores coated with a layer of bovine type I collagen matrix. The lower chamber was filled with DMEM containing 10% FBS as the chemoattractant. The assembled chambers were then placed in the CO_2_ incubator for 20 h. Cells that passed through the membrane pores were lysed and quantitated using a fluorescence dye-containing solution and a fluorescence plate reader. Statistical significance was analyzed using the standard Student's *t*-test. *P*-values <0.05 were considered statistically significant.

### Wound-healing assay

Cells were grown to confluence in DMEM containing 0.5% FBS in 6-well plates. Scratch wounds were made using 10-μL sterile pipette tips. The cells were incubated in DMEM containing 10% FBS for the indicated times. Images were captured using an Evolution MP camera (Media Cybernetics, Rockville, MD, USA).

### 3D cell proliferation assay

3D cell proliferation *in vitro* was measured using the CellTiter-Glo^®^ 3D cell viability assay (Promega) according to the manufacturer's instructions [55]. Briefly, the cells were plated (500 cells/100 μL/well) in triplicate in 96-well ultra low attachment plates (Costar Corning, Schiphol-Rijk, Netherlands) with DMEM containing 10% FBS. The 3D cell viability was measured every 24 h for 72 h. The CellTiter-Glo^®^ 3D reagent was added into wells in a 1:1 dilution (100 μL volume in well: 100 μL of reagent) and then the solutions were mixed well by pipetting. After incubation for 30 min at 37°C, the luminescent signal was read using an EnSpire multi-plate reader (Perkin Elmer, PerkinElmer, Waltham, MA, USA). Images were captured using an Evolution MP camera (Media Cybernetics). In addition, to investigate the influence of the antibodies on the 3D cell proliferation, the cells were plated as described above with or without the antibodies (30 μg/ml). The proliferation rate was calculated relative to the signal at 0 h.

### ADCC

ADCC was examined using the ^51^Cr-release assay. Effector cells were prepared as previously described [36]. Mouse splenocytes were harvested from spleens of six-week-old male SCID mice. Spleens were homogenized in RPMI 1640 and centrifuged. To deplete red blood cells, the cell pellet was suspended in red blood cell lysis buffer (Sigma-Aldrich). After washing and re-suspension in 10% FBS/RPMI1640, splenocytes were used as effector cells. Target cells were incubated with 0.1 μCi of [^51^Cr]sodium chromate at 37°C for 1 h. After three washes with 10% FBS/RPMI1640, ^51^Cr-labeled target cells were placed in 96-well plates in triplicate. Effector cells and antibodies (10 μg/ml) were added to the plates (E/T ratio = 100). After 6, 12, and 18 hrs of incubation, ^51^Cr release was measured in the supernatant (100 μL) from each well using a gamma counter (PerkinElmer). The percentage of cytotoxicity was calculated using the following formula: % specific lysis = (E – S)/(M − S) × 100, where E is the ^51^Cr release in the test sample, S is the spontaneous release, and M is the maximum release. Statistical significance was analyzed using the standard Student's *t*-test. *P*-values <0.05 were considered statistically significant.

### CDC

CDC was evaluated using the ^51^Cr release assay. Target cells were incubated with [^51^Cr]sodium chromate (0.1 μCi) for 1 h at 37°C. The cells were then washed with 10% FBS/RPMI1640. The ^51^Cr-labeled cells were incubated with a baby rabbit complement Cedarlane (Ontario, Canada) at a dilution of 1:4 in the presence of antibodies (10 μg/mL) for 6 h in 96-well plates. After the incubation, ^51^Cr radioactivity was measured in the supernatants using a gamma counter. The percent cytotoxicity was calculated using the following formula: % specific lysis = (E – S)/(M − S) × 100, where E is the release in the test sample, S is the spontaneous release, and M is the maximum release. Statistical significance was analyzed using the standard Student's *t*-test. *P*-values < 0.05 were considered statistically significant.

### Antitumor activity of anti-PODXL antibodies

Five-week-old female BALB/c nude mice were purchased from Charles River and used in experiments at 7 weeks of age. Cells (0.3 mL of 1.33 × 10^8^ /mL in DMEM) were mixed with 0.5 mL of BD Matrigel Matrix Growth Factor Reduced (BD Biosciences). A 100-μL suspension (containing 5 × 10^6^ cells) was injected subcutaneously into the right flanks of nude mice. After 1 day, 100 μg or 500 μg of 47-mG_2a_, 47-mG_2a_-f, or mouse IgG in 200 μL PBS were injected into the peritoneal cavity of each mouse. Additional antibodies were injected once weekly for several weeks. The tumor diameter and tumor volume were determined as previously described. The mice were euthanized 15, 16, 20, or 21 days after cell implantation. All data were expressed as the mean ± SEM. Statistical analysis was performed using the Tukey-Kramer test. *P*-values < 0.05 was considered statistically significant.

## SUPPLEMENTARY MATERIALS FIGURES AND TABLE





## Abbreviations

AAL: Aleuria aurantia lectin
ADCC: Antibody-dependent cellular cytotoxicity
CDC: Complement-dependent cytotoxicity
DFS: Disease-free survival
DMEM: Dulbecco's Modified Eagle's Medium
DSS: Disease-specific survival
EGFR: Epidermal growth factor receptor
ELISA: Enzyme-linked immunosorbent assay
HE: Hematoxylin & eosin
HNC: Head and neck cancer
HNSCC: Head and neck squamous cell carcinoma
LCA: Lens culinaris agglutinin
OS: Overall survival
OSCC: Oral squamous cell carcinoma
PhoSL: Pholiota squarrosa lectin
PSA: Pisum sativum agglutinin
R/M: Recurrent and/or metastatic
