# Supplementary material for: Anti-podocalyxin antibody exerts antitumor effects via antibody-dependent cellular cytotoxicity in mouse xenograft models of oral squamous cell carcinoma
Source: Oncotarget. 2018 Apr 27;9(32):22480–97. doi: 10.18632/oncotarget.25132 (PMC5976479; doi:10.18632/oncotarget.25132)
Supplement: Supplementary file 1 [file oncotarget-09-22480-s001.pdf]

Anti-podocalyxin antibody exerts antitumor effects via antibody-dependent cellular cytotoxicity in mouse xenograft models of oral squamous cell carcinoma

SUPPLEMENTARY MATERIALS

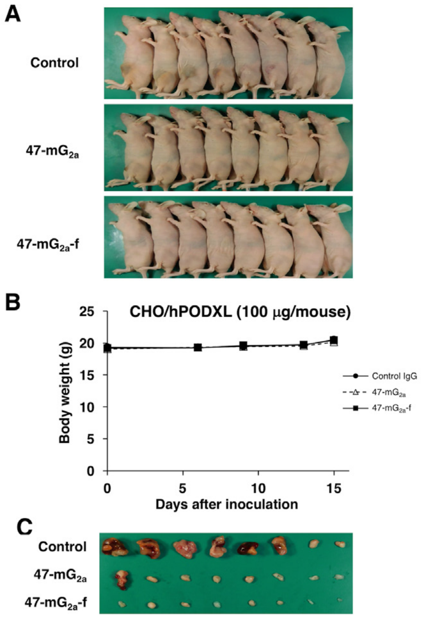

Supplementary Figure 1: Antitumor activity of 47-mG$_{2a}$ and 47-mG$_{2a}$-f against CHO/hPODXL xenografts (100 µg/day; 5 mg/kg). **(A)** Comparison of the tumor size and tumor incidence of CHO/hPODXL xenograft in nude mice (day 16). **(B)** Body weight. The values are means ± SEM **(C)** Comparison of the tumor size (day 16).

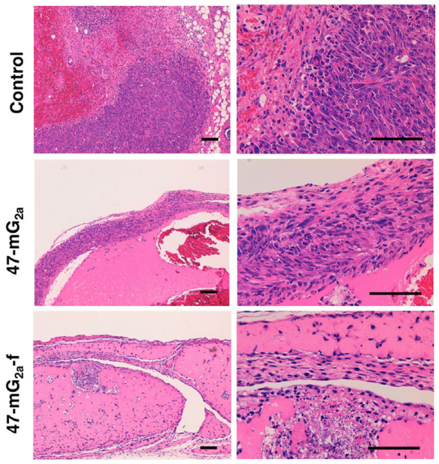

Supplementary Figure 2: Hematoxylin & eosin staining of CHO/hPODXL xenografts (100 µg/day; 5 mg/kg). Scale bar = 100 µm.

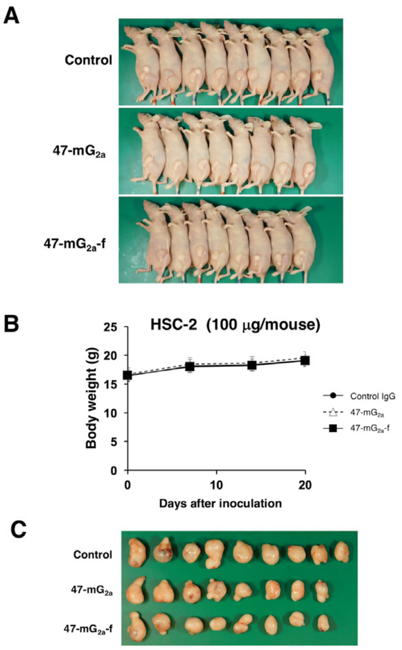

Supplementary Figure 3: Antitumor activity of 47-mG$_{2a}$ and 47-mG$_{2a}$-f against HSC-2 xenografts (100 µg/day; 5 mg/kg). **(A)** Comparison of the tumor size and tumor incidence of HSC-2 xenograft in nude mice (day 20). **(B)** Body weight. The values are means ± SEM **(C)** Comparison of the tumor size (day 20).

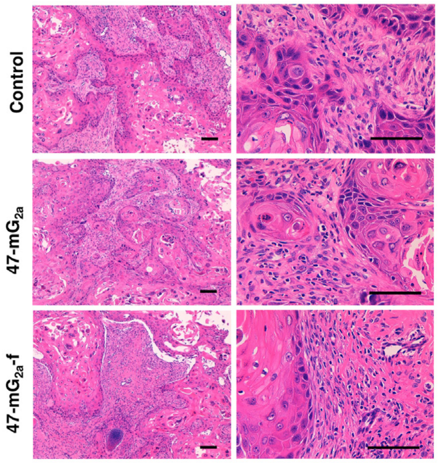

Supplementary Figure 4: Hematoxylin & eosin staining of HSC-2 xenografts (100 µg/day; 5 mg/kg). Scale bar = 100 µm.

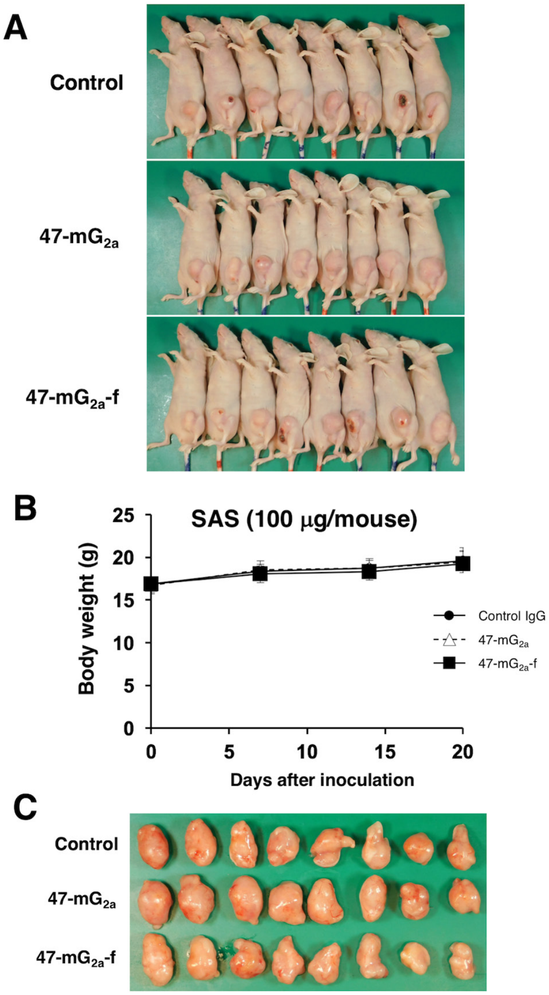

Supplementary Figure 5:Antitumor activity of 47-mG$_{2a}$ and 47-mG$_{2a}$-f against SAS xenografts (100 µg/day; 5 mg/kg). (**A)** Comparison of the tumor size and tumor incidence of SAS xenograft in nude mice (day 20). **(B)** Body weight. The values are means ± SEM **(C)** Comparison of the tumor size (day 20).

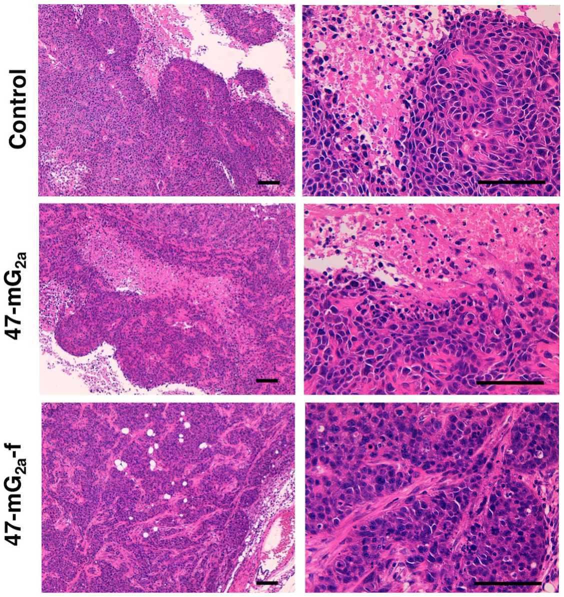

Supplementary Figure 6: Hematoxylin & eosin staining of SAS xenografts (100 µg/day; 5 mg/kg). Scale bar = 100 µm.

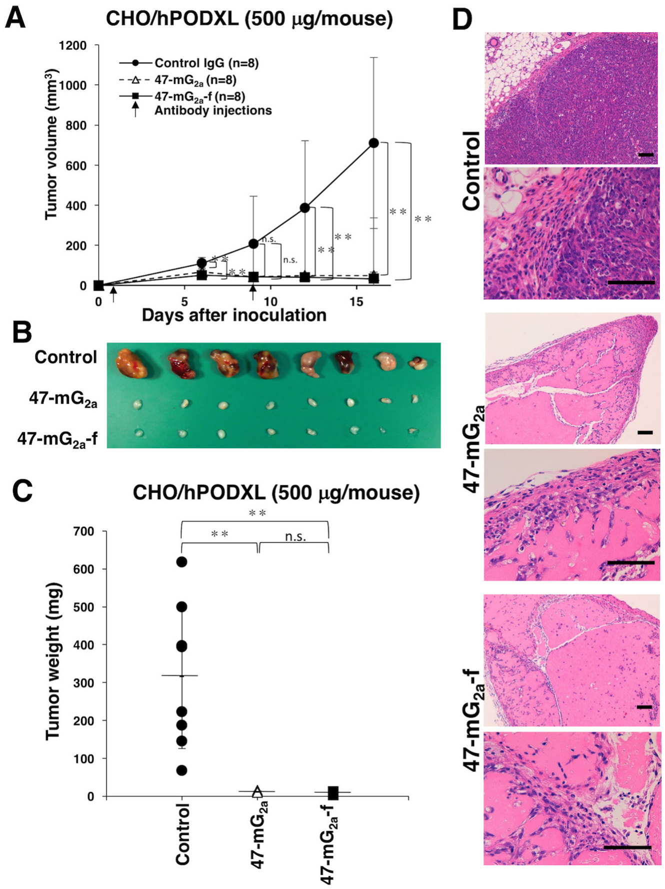

Supplementary Figure 7: Antitumor activity of 47-mG$_{2a}$ and 47-mG$_{2a}$-f against CHO/hPODXL xenografts (500 µg/day; 25 mg/kg). **(A)** Tumor volume of CHO/hPODXL xenografts. CHO/hPODXL cells were injected subcutaneously into female nude mice. The indicated antibodies (500 µg/day; 25 mg/kg) were administered intraperitoneally 1 and 9 days after cancer cell inoculation. The tumor volume was measured at the indicated time points. The values are means ± SEM. **(B)** Comparison of tumor size (day 16). **(C)** Tumor weight of CHO/hPODXL xenografts (day 16). **(D)** Hematoxylin & eosin staining of CHO/hPODXL xenografts. An asterisk indicates statistical significance ($^{**}P < 0.01$, Tukey-Kramer's test). Scale bar = 100 µm.

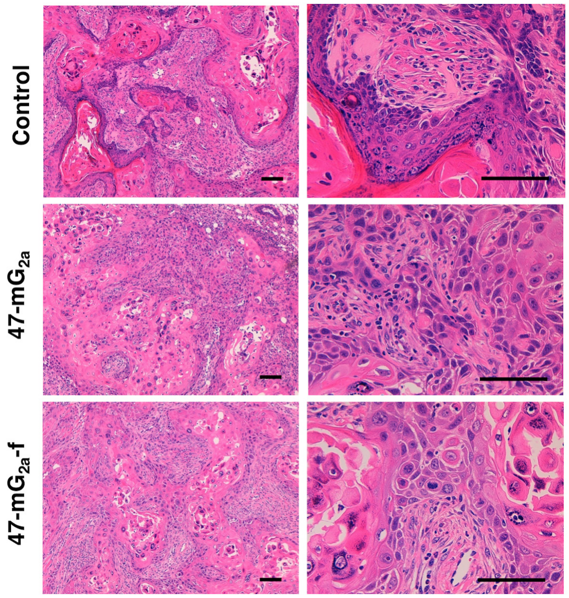

Supplementary Figure 8: Antitumor activity of 47-mG$_{2a}$ and 47-mG$_{2a}$-f against HSC-2 xenografts (500 µg/day; 25 mg/kg). Hematoxylin & eosin staining of HSC-2 xenografts.

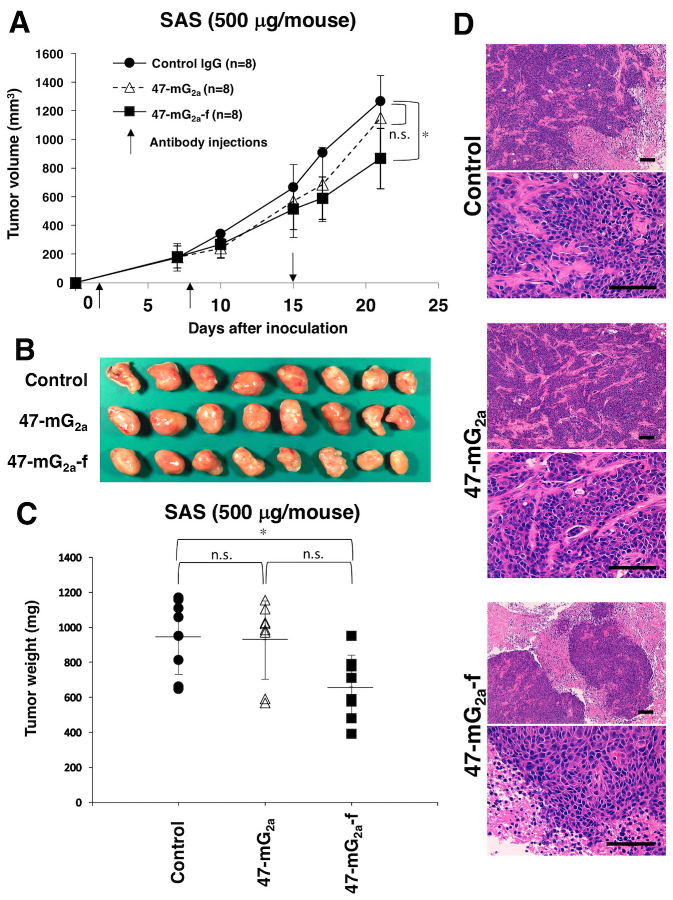

Supplementary Figure 9: Antitumor activity of 47-mG$_{2a}$ and 47-mG$_{2a}$-f against SAS xenografts (500 µg/day; 25 mg/kg). **(A)** Tumor volume of SAS xenografts. SAS cells were injected subcutaneously into female nude mice. The indicated antibodies (500 µg/day; 25 mg/kg) were administered intraperitoneally 1, 8, and 15 days after cancer cell inoculation. The tumor volume was measured at the indicated time points. The values are means ± SEM. **(B)** Comparison of tumor size (day 21). **(C)** Tumor weight of SAS xenografts (day 21). **(D)** Hematoxylin & eosin staining of SAS xenografts. An asterisk indicates statistical significance ($^*P < 0.05$, Tukey-Kramer's test). Scale bar = 100 µm.

Supplementary Table 1: Immunohistochemical analysis by anti-PODXL mAbs against OSCC.

See Supplemenrtary File 1
