# Supplementary material for: Anti-podocalyxin antibody exerts antitumor effects via antibody-dependent cellular cytotoxicity in mouse xenograft models of oral squamous cell carcinoma
Source: Oncotarget. 2018 Apr 27;9(32):22480–97. doi: 10.18632/oncotarget.25132 (PMC5976479; doi:10.18632/oncotarget.25132)
Supplement: Supplementary file 2 [file oncotarget-09-22480-s002.doc]

Supplementary table 1. Immunohistochemical analysis by anti-PODXL mAbs against OSCC.

| No. | Age | Sex | Organ | Pathology diagnosis | Differentiation | Type | TNM | PcMab-47  (5 μg/ml) | 47-mG_2a_  (0.5 μg/ml) |
| --- | --- | --- | --- | --- | --- | --- | --- | --- | --- |
| 1 | 61 | M | Tongue | SCC | Well | Malignant | N/D | - | 1+ |
| 2 | 57 | F | Tongue | SCC | Well | Malignant | N/D | - | 2+ |
| 3 | 67 | F | Tongue | SCC | Well | Malignant | N/D | - | 1+ |
| 4 | 59 | M | Tongue | SCC | Well | Malignant | N/D | - | 2+ |
| 5 | 47 | F | Tongue | SCC | Well | Malignant | N/D | - | 1+ |
| 6 | 62 | F | Tongue | SCC | N/D | Malignant | N/D | - | 1+ |
| 7 | 47 | F | Tongue | SCC | Well | Malignant | N/D | 1+ | 1+ |
| 8 | 51 | M | Tongue | SCC | Well | Malignant | N/D | 1+ | 1+ |
| 9 | 62 | M | Tongue | SCC | Well | Malignant | N/D | - | 2+ |
| 10 | 53 | F | Tongue | SCC | Well | Malignant | N/D | 1+ | 1+ |
| 11 | 50 | M | Tongue | SCC | Well | Malignant | N/D | - | 1+ |
| 12 | 76 | M | Tongue | SCC | Well | Malignant | N/D | 1+ | 1+ |
| 13 | 55 | M | Tongue | SCC | Well | Malignant | N/D | - | 1+ |
| 14 | 57 | F | Tongue | SCC | Well | Malignant | N/D | - | 1+ |
| 15 | 61 | M | Tongue | SCC | Well | Malignant | N/D | 1+ | 2+ |
| 16 | 50 | F | Tongue | SCC | Well | Malignant | N/D | 1+ | 1+ |
| 17 | 54 | M | Tongue | SCC | Well | Malignant | N/D | 1+ | 2+ |
| 18 | 62 | M | Tongue | SCC | Well | Malignant | N/D | - | 1+ |
| 19 | 55 | F | Tongue | SCC | Well | Malignant | N/D | 1+ | 2+ |
| 20 | 63 | F | Tongue | SCC | Well | Malignant | N/D | 1+ | 1+ |
| 21 | 56 | M | Tongue | SCC | Well | Malignant | N/D | - | 1+ |
| 22 | 45 | F | Tongue | SCC | Well | Malignant | N/D | 1+ | 1+ |
| 23 | 50 | M | Tongue | SCC | Well | Malignant | N/D | 3+ | 3+ |
| 24 | 46 | F | Tongue | SCC | Poorly | Malignant | N/D | 1+ | 1+ |
| 25 | 48 | F | Tongue | SCC | Moderately | Malignant | N/D | 1+ | 2+ |
| 26 | 67 | F | Tongue | SCC | N/D | Malignant | N/D | - | 1+ |
| 27 | 64 | M | Tongue | SCC | Poorly | Malignant | N/D | - | 1+ |
| 28 | 50 | F | Tongue | SCC | Moderately | Malignant | N/D | 1+ | 1+ |
| 29 | 63 | M | Tongue | SCC | Moderately | Malignant | N/D | 1+ | 1+ |
| 30 | 46 | F | Tongue | SCC | Well | Malignant | N/D | 1+ | 1+ |
| 31 | 35 | F | Tongue | SCC | Moderately | Malignant | N/D | 1+ | 1+ |
| 32 | 55 | M | Tongue | SCC | Poorly | Malignant | N/D | 3+ | 3+ |
| 33 | 49 | M | Tongue | SCC | Poorly | Malignant | N/D | - | 2+ |
| 34 | 61 | M | Tongue | SCC | Moderately | Malignant | N/D | 1+ | 3+ |
| 35 | 53 | M | Tongue | SCC | Moderately | Malignant | N/D | - | 1+ |
| 36 | 51 | F | Tongue | SCC | Poorly | Malignant | N/D | 2+ | 3+ |
| 37 | 73 | M | Tongue | SCC | Poorly | Malignant | N/D | - | 1+ |
| 38 | 61 | M | Tongue | SCC | Poorly | Malignant | N/D | 1+ | 2+ |
| 39 | 78 | M | Tongue | SCC | Well | Malignant | T2N0M0 | 1+ | 2+ |
| 40 | 51 | F | Tongue | SCC | Well | Malignant | T4N0M0 | 1+ | 2+ |
| 41 | 75 | F | Tongue | SCC | Well | Malignant | T2N0M0 | - | 1+ |
| 42 | 69 | M | Tongue | SCC | Well | Malignant | T3N0M0 | 1+ | 2+ |
| 43 | 56 | F | Tongue | SCC | Well | Malignant | T2N0M0 | 1+ | 1+ |
| 44 | 35 | F | Tongue | SCC | Well | Malignant | T2N0M0 | 1+ | 1+ |
| 45 | 39 | F | Tongue | SCC | Well | Malignant | T1N0M0 | 1+ | 1+ |
| 46 | 64 | M | Tongue | SCC | Well | Malignant | T1N0M0 | - | - |
| 47 | 63 | M | Tongue | SCC | Well | Malignant | T1N0M0 | - | 1+ |
| 48 | 77 | F | Tongue | SCC | Moderately | Malignant | T1N0M0 | 1+ | 2+ |
| 49 | 41 | F | Tongue | SCC | Well | Malignant | T2N0M0 | - | 1+ |
| 50 | 53 | M | Tongue | SCC | Well | Malignant | T2N0M0 | 1+ | 2+ |
| 51 | 50 | M | Tongue | SCC | Well | Malignant | T3N0M0 | 1+ | 1+ |
| 52 | 36 | F | Tongue | SCC | Moderately | Malignant | T1N0M0 | 1+ | 2+ |
| 53 | 58 | M | Tongue | SCC | Well | Malignant | T1N0M0 | 1+ | 3+ |
| 54 | 63 | F | Tongue | SCC | Well | Malignant | T1N0M0 | 1+ | 2+ |
| 55 | 55 | F | Tongue | SCC | Moderately | Malignant | T2N0M0 | 1+ | 1+ |
| 56 | 76 | M | Tongue | SCC | Well | Malignant | T1N0M0 | 1+ | 1+ |
| 57 | 50 | F | Tongue | SCC | Well | Malignant | T2N0M0 | 1+ | 1+ |
| 58 | 44 | M | Tongue | SCC | Well | Malignant | T2N1M0 | 2+ | 2+ |
| 59 | 53 | F | Tongue | SCC | Well | Malignant | T1N0M0 | 1+ | 1+ |
| 60 | 60 | M | Tongue | SCC | N/D | Malignant | T1N0M0 | 1+ | 1+ |
| 61 | 55 | M | Tongue | SCC | Well | Malignant | T1N0M0 | 1+ | 2+ |
| 62 | 61 | M | Tongue | SCC | Well | Malignant | T1N0M0 | 1+ | 2+ |
| 63 | 55 | M | Tongue | SCC | Well | Malignant | T1N0M0 | - | 1+ |
| 64 | 59 | M | Tongue | SCC | Well | Malignant | T2N0M0 | 3+ | 3+ |
| 65 | 46 | F | Tongue | SCC | Well | Malignant | T2N0M0 | 1+ | 2+ |
| 66 | 45 | F | Tongue | SCC | Well | Malignant | T2N0M0 | 1+ | 1+ |
| 67 | 61 | M | Tongue | SCC | Well | Malignant | T2N0M0 | 1+ | 2+ |
| 68 | 48 | F | Tongue | SCC | Well | Malignant | T2N0M0 | - | 1+ |
| 69 | 52 | F | Tongue | SCC | Well | Malignant | T1N0M0 | 1+ | 1+ |
| 70 | 64 | M | Tongue | SCC | Well | Malignant | T2N0M0 | 1+ | 3+ |
| 71 | 46 | F | Tongue | SCC | Well | Malignant | T2N0M0 | - | 1+ |
| 72 | 48 | F | Tongue | SCC | Well | Malignant | T1N0M0 | 1+ | 1+ |
| 73 | 80 | M | Tongue | SCC | Well | Malignant | T1N0M0 | 1+ | 1+ |
| 74 | 49 | M | Tongue | SCC | N/D | Malignant | T1N0M0 | - | 1+ |
| 75 | 60 | M | Tongue | SCC | Well | Malignant | T2N0M0 | 1+ | 1+ |
| 76 | 57 | M | Tongue | SCC | Well | Malignant | T1N0M0 | 3+ | 2+ |
| 77 | 45 | M | Tongue | SCC | Well | Malignant | T2N0M0 | 1+ | 1+ |
| 78 | 47 | F | Tongue | SCC | Well | Malignant | T2N0M0 | 1+ | 1+ |
| 79 | 37 | M | Tongue | SCC | Well | Malignant | T2N1M0 | - | - |
| 80 | 60 | M | Tongue | SCC | Moderately | Malignant | T2N0M0 | 1+ | 1+ |
| 81 | 40 | F | Tongue | SCC | Poorly | Malignant | T2N0M0 | - | 1+ |
| 82 | 49 | M | Tongue | SCC | Moderately | Malignant | T1N0M0 | 1+ | 2+ |
| 83 | 50 | M | Tongue | SCC | Poorly | Malignant | T2N0M0 | 1+ | 2+ |
| 84 | 60 | M | Tongue | SCC | Poorly | Malignant | T1N0M0 | 1+ | 1+ |
| 85 | 56 | F | Tongue | SCC | Poorly | Malignant | T2N0M0 | - | 1+ |
| 86 | 77 | M | Tongue | SCC | Poorly | Malignant | T2N0M0 | 2+ | 2+ |
| 87 | 56 | M | Tongue | SCC | Moderately | Malignant | T2N1M0 | 1+ | 2+ |
| 88 | 35 | M | Tongue | SCC | Well | Malignant | T2N0M0 | 1+ | 1+ |
| 89 | 58 | M | Tongue | SCC | Moderately | Malignant | T2N1M0 | 1+ | 1+ |
| 90 | 61 | M | Tongue | SCC | Well | Malignant | T2N0M0 | 1+ | 1+ |
| 91 | 45 | M | Tongue | SCC | Well | Malignant | T2N0M0 | 1+ | 1+ |
| 92 | 72 | M | Tongue | SCC | Poorly | Malignant | T2N0M0 | 1+ | 1+ |
| 93 | 46 | F | Gums | SCC | Well | Malignant | T2N2M0 | 1+ | 1+ |
| 94 | 61 | M | Tongue | SCC | Moderately | Malignant | T2N1M0 | 1+ | 2+ |
| 95 | 54 | M | Tongue | SCC | Poorly | Malignant | T2N1M0 | 1+ | 1+ |
| 96 | 58 | M | Tongue | SCC | Moderately | Malignant | T2N1M0 | 1+ | 1+ |
| 97 | 64 | F | Tongue | SCC | Well | Malignant | T2N0M0 | 1+ | 1+ |
| 98 | 56 | M | Gums | SCC | Moderately | Malignant | T3N0M0 | 2+ | 1+ |
| 99 | 54 | M | Gums | SCC | Moderately | Malignant | T1N0M0 | 1+ | 1+ |
| 100 | 59 | F | Tongue | SCC | Moderately | Malignant | T2N0M0 | 1+ | 1+ |
| 101 | 57 | M | Tongue | SCC | Well | Malignant | T1N2M0 | 2+ | 2+ |
| 102 | 5 | M | Tongue | SCC | Moderately | Malignant | T2N2M0 | - | 1+ |
| 103 | 43 | M | Tongue | SCC | Poorly | Malignant | T3N0M0 | - | 1+ |
| 104 | 61 | M | Tongue | SCC | Well | Malignant | T2N1M0 | 1+ | 1+ |
| 105 | 64 | M | Mouth floor | SCC | Well | Malignant | N/D | 1+ | 1+ |
| 106 | 57 | M | Tongue | SCC | Moderately | Malignant | T1N0M0 | 1+ | 1+ |
| 107 | 67 | M | Tongue | SCC | Well | Malignant | T4N1M0 | 1+ | 1+ |
| 108 | 50 | M | Tongue | SCC | Moderately | Malignant | T1N1M0 | 1+ | 1+ |
| 109 | 65 | M | Tongue | SCC | Well | Malignant | T2N2M0 | - | 1+ |
| 110 | 71 | M | Tongue | SCC | Well | Malignant | T3N0M0 | 1+ | 1+ |
| 111 | 57 | M | Tongue | SCC | Poorly | Malignant | T3N0M0 | 2+ | 2+ |
| 112 | 54 | M | Soft palate | SCC | Moderately | Malignant | T3N0M0 | 1+ | 1+ |
| 113 | 53 | M | Tongue | SCC | Well | Malignant | T3N1M0 | 1+ | 1+ |
| 114 | 59 | M | Gums | SCC | Well | Malignant | T1N0M0 | 2+ | 2+ |
| 115 | 58 | F | Gums | SCC | Well | Malignant | T2N0M0 | 2+ | 2+ |
| 116 | 53 | M | Tongue | SCC | Well | Malignant | T1N0M0 | 1+ | 1+ |
| 117 | 57 | F | Tongue | SCC | Well | Malignant | T2N1M0 | 1+ | 1+ |
| 118 | 36 | M | Tongue | SCC | Well | Malignant | T1N0M0 | 1+ | 1+ |
| 119 | 44 | M | Buccal mucosa | SCC | Moderately | Malignant | T3N0M0 | 1+ | 1+ |
| 120 | 77 | M | Tongue | SCC | Well | Malignant | T3N0M0 | - | 1+ |
| 121 | 43 | F | Tongue | SCC | Poorly | Malignant | T3N0M0 | 1+ | 1+ |
| 122 | 80 | M | Gums | SCC | Moderately | Malignant | T1N1M0 | 1+ | 1+ |
| 123 | 69 | M | Left mandible | SCC | Well | Malignant | T2N1M0 | 1+ | 1+ |
| 124 | 76 | F | Gums | SCC | N/D | Malignant | T2N1M0 | 1+ | 1+ |
| 125 | 76 | F | Tongue | SCC | Well | Malignant | T3N0M0 | - | 1+ |
| 126 | 65 | F | Tongue | SCC | Well | Malignant | T4N0M0 | 1+ | 1+ |
| 127 | 42 | M | Mouth floor | SCC | Well | Malignant | T1N0M0 | 1+ | 1+ |
| 128 | 60 | F | Tongue | SCC | Well | Malignant | T1N0M0 | 2+ | 1+ |
| 129 | 58 | M | Tongue | SCC | Moderately | Malignant | T3N1M0 | - | 1+ |
| 130 | 52 | M | Tongue | SCC | Well | Malignant | T1N0M0 | 1+ | 1+ |
| 131 | 60 | F | Tongue | SCC | Well | Malignant | T2N0M0 | 1+ | 1+ |
| 132 | 43 | F | Tongue | SCC | Well | Malignant | T3N0M0 | - | 1+ |
| 133 | 40 | F | Tongue | SCC | Well | Malignant | T2N0M0 | 1+ | 1+ |
| 134 | 49 | M | Tongue | SCC | Well | Malignant | T1N0M0 | 1+ | 1+ |
| 135 | 69 | M | Pharynx | SCC | Well | Malignant | T4N2M0 | 1+ | 1+ |
| 136 | 54 | M | Tongue | SCC | Moderately | Malignant | T2N0M0 | 1+ | 1+ |
| 137 | 63 | F | Gums | SCC | Well | Malignant | T2N1M0 | 2+ | 2+ |
| 138 | 69 | F | Gums | SCC | Well | Malignant | T2N0M0 | 2+ | 2+ |
| 139 | 55 | M | Mouth floor | SCC | Well | Malignant | T2N1M1 | 1+ | 1+ |
| 140 | 63 | F | Tongue | SCC | Well | Malignant | T2N0M0 | 1+ | 1+ |
| 141 | 47 | M | Tongue | SCC | Well | Malignant | T2N1M0 | 1+ | 1+ |
| 142 | 49 | M | Tongue | SCC | Moderately | Malignant | T2N0M0 | 1+ | 1+ |
| 143 | 61 | M | Tongue | SCC | Well | Malignant | T1N0M0 | 1+ | 1+ |
| 144 | 71 | M | Palate | SCC | Moderately | Malignant | T3N1M0 | 2+ | 1+ |
| 145 | 47 | M | Tongue | SCC | Well | Malignant | T2N1M0 | 1+ | 1+ |
| 146 | 60 | M | Mouth floor | SCC | Poorly | Malignant | T2N0M0 | 1+ | 1+ |
| 147 | 58 | M | Tongue | SCC | Moderately | Malignant | T3N0M0 | 2+ | 2+ |
| 148 | 49 | F | Tongue | SCC | Poorly | Malignant | T3N0M0 | 2+ | 1+ |
| 149 | 67 | M | Tongue | SCC | Well | Malignant | T2N0M0 | 3+ | 3+ |
| 150 | 64 | M | Tongue | SCC | Moderately | Malignant | T2N0M0 | 1+ | 1+ |
| 151 | 55 | M | Tongue | SCC | Moderately | Malignant | T2N0M0 | 2+ | 2+ |
| 152 | 51 | M | Tongue | SCC | Well | Malignant | T1N0M0 | 1+ | 1+ |
| 153 | 73 | M | Tongue | SCC | Well | Malignant | T1N0M0 | 2+ | 2+ |
| 154 | 34 | F | Tongue | SCC | Well | Malignant | T1N0M0 | - | 1+ |
| 155 | 29 | F | Tongue | SCC | Well | Malignant | T1N0M0 | 2+ | 2+ |
| 156 | 43 | F | Tongue | SCC | Moderately | Malignant | T2N0M0 | 3+ | 3+ |
| 157 | 29 | F | Tongue | SCC | Well | Malignant | T1N0M0 | 1+ | 1+ |
| 158 | 52 | F | Tongue | SCC | Moderately | Malignant | T1N0M0 | 1+ | 1+ |
| 159 | 39 | M | Tongue | SCC | Well | Malignant | T2N1M0 | 1+ | 1+ |
| 160 | 49 | M | Tongue | SCC | Well | Malignant | T2N2bM0 | 1+ | 1+ |
| 161 | 67 | M | Tongue | SCC | Well | Malignant | T2N0M0 | 2+ | 1+ |
| 162 | 69 | M | Tongue | SCC | Well | Malignant | T1N0M0 | 1+ | 1+ |
| 163 | 35 | F | Tongue | SCC | Moderately | Malignant | T2N0M0 | 1+ | 1+ |
| 164 | 55 | F | Tongue | SCC | Well | Malignant | T2N0M0 | 1+ | 1+ |
| 165 | 65 | M | Tongue | SCC | Moderately | Malignant | T3N2aM0 | 2+ | 2+ |
| 166 | 75 | M | Tongue | SCC | Well | Malignant | T2N1M0 | 2+ | 2+ |
| 167 | 64 | M | Tongue | SCC | Well | Malignant | T2N1M0 | 2+ | 2+ |
| 168 | 78 | M | Tongue | SCC | Well | Malignant | T2N0M0 | 1+ | 1+ |
| 169 | 50 | F | Tongue | SCC | Well | Malignant | T2N0M0 | 2+ | 2+ |
| 170 | 64 | M | Tongue | SCC | Well | Malignant | T2N0M0 | - | 1+ |
| 171 | 48 | M | Tongue | SCC | Well | Malignant | T1N0M0 | 3+ | 3+ |
| 172 | 64 | M | Tongue | SCC | Moderately | Malignant | T2N0M0 | 3+ | 3+ |
| 173 | 50 | M | Tongue | SCC | Moderately | Malignant | T3N1M0 | 3+ | 3+ |
| 174 | 65 | M | Tongue | SCC | Moderately | Malignant | T3N0M0 | 2+ | 3+ |
| 175 | 44 | F | Tongue | SCC | Moderately | Malignant | T2N0M0 | 1+ | 1+ |
| 176 | 62 | M | Tongue | SCC | Moderately | Malignant | T1N0M0 | 1+ | 1+ |
| 177 | 58 | F | Tongue | SCC | Well | Malignant | T2N0M0 | 2+ | 3+ |
| 178 | 57 | F | Tongue | SCC | Poorly | Malignant | T2N0M0 | 1+ | 1+ |
| 179 | 38 | M | Tongue | SCC | Well | Malignant | T3N0M0 | 1+ | 1+ |
| 180 | 43 | M | Tongue | SCC | Moderately | Malignant | T1N2bM0 | 1+ | 1+ |
| 181 | 75 | M | Tongue | SCC | Moderately | Malignant | T2N0M0 | 3+ | 2+ |
| 182 | 63 | M | Tongue | SCC | Well | Malignant | T2N2bM0 | 3+ | 3+ |
| 183 | 71 | M | Tongue | SCC | Well | Malignant | T1N0M0 | - | - |
| 184 | 64 | M | Tongue | SCC | Well | Malignant | T2N0M0 | 1+ | 2+ |
| 185 | 52 | M | Tongue | SCC | Well | Malignant | T1N0M0 | 2+ | 2+ |
| 186 | 48 | M | Tongue | SCC | Well | Malignant | T2N1M0 | 2+ | 2+ |
| 187 | 78 | M | Tongue | SCC | Moderately | Malignant | T2N0M0 | 2+ | 1+ |
| 188 | 30 | M | Tongue | SCC | Moderately | Malignant | T2N0M0 | 2+ | 2+ |
| 189 | 66 | M | Tongue | SCC | Moderately | Malignant | T3N1M0 | 2+ | 1+ |
| 190 | 59 | M | Tongue | SCC | Moderately | Malignant | T2N2cM0 | 1+ | - |
| 191 | 59 | M | Tongue | SCC | Moderately | Malignant | T2N0M0 | 2+ | 1+ |
| 192 | 72 | M | Tongue | SCC | Well | Malignant | T1N1M0 | 1+ | 1+ |
| 193 | 73 | M | Tongue | SCC | Moderately | Malignant | T3N0M0 | 1+ | 1+ |
| 194 | 63 | M | Tongue | SCC | Moderately | Malignant | T2N0M0 | 1+ | 1+ |
| 195 | 30 | M | Tongue | SCC | Well | Malignant | T2N0M0 | 1+ | 2+ |
| 196 | 50 | F | Tongue | SCC | Well | Malignant | T2N0M0 | 2+ | 2+ |
| 197 | 78 | M | Tongue | SCC | Moderately | Malignant | T3N2aM1 | 1+ | 1+ |
| 198 | 75 | M | Tongue | SCC | Poorly | Malignant | T2N2cM0 | 2+ | 2+ |
| 199 | 74 | M | Tongue | SCC | Moderately | Malignant | T2N2bM0 | 2+ | 2+ |
| 200 | 54 | F | Tongue | SCC | Moderately | Malignant | T2N1M0 | 1+ | 1+ |
| 201 | 73 | M | Tongue | SCC | Moderately | Malignant | T3N2bM0 | 1+ | 1+ |

N/D, not determined.

The intensity of staining was evaluated as -, 1+, 2+, 3+
